# DNA methylation variations of DNA damage response correlate survival and local immune status in melanomas

**DOI:** 10.1002/iid3.1331

**Published:** 2024-09-10

**Authors:** Min Wang, Xiao‐dong Zhang, Han‐qing Yang, Yang Li, Wen‐mei Chen, An‐an Yin

**Affiliations:** ^1^ Department of Burns and Plastic Surgery Changzhou Wujin People's Hospital Changzhou China; ^2^ Department of Plastic and Reconstructive Surgery, Xijing Hospital Fourth Military Medical University Xi'an China; ^3^ Shaanxi Provincial Key Laboratory of Clinic Genetics Fourth Military Medical University Xi'an China

**Keywords:** DNA damage response, DNA methylation, immunity, melanoma, prognosis

## Abstract

**Aim:**

We aimed to explore the impact of DNA methylation alterations on the DNA damage response (DDR) in melanoma prognosis and immunity.

**Material & methods:**

Different melanoma cohorts with molecular and clinical data were included.

**Results:**

Hierarchical clustering utilizing different combinations of DDR‐relevant CpGs yielded distinct melanoma subtypes, which were characteristic of different prognoses, transcriptional function profiles of DDR, and immunity and immunotherapy responses but were associated with similar tumor mutation burdens. We then constructed and validated a clinically applicable 4‐CpG risk‐score signature for predicting survival and immunotherapy response.

**Conclusion:**

Our study describes the close interrelationship among DNA methylation, DDR machinery, local tumor immune status, melanoma prognosis, and immunotherapy response.

## Graphical abstract

The present study proposed a series of DNA damage response (DDR) DNA methylation‐based subtypes of melanomas with distinct prognosis, transcriptional function profiles of DDR and immunity, and immunotherapy response. Those novel epigenetic DDR classification schemes for melanomas not only provide ideal models for illustrating the interrelationship among DNA methylation, DDR function, local tumor immunity, immunotherapy efficacy, and cancer prognosis but also provide useful information for predicting immunotherapy response and guiding the combinatorial use of epigenetic therapy, DDR inhibitors, DNA‐damaging agents and immunotherapy.

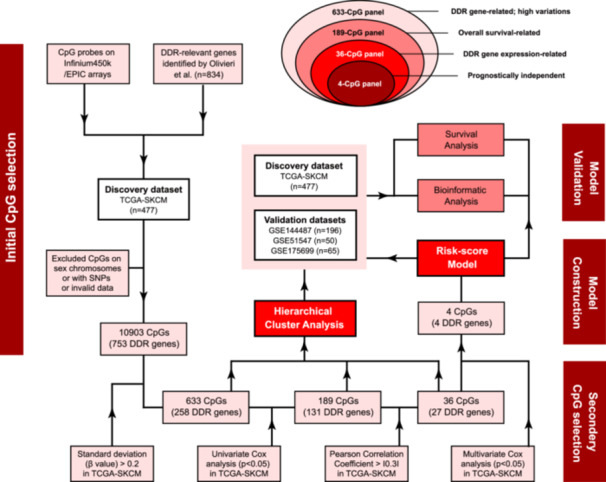

## 1 PLAIN LANGUAGE SUMMARY

DNA damage response (DDR) has been increasingly regarded as a central player in tumor immunity. In our study, hierarchical clustering utilizing DNA methylation alterations of DDR genes yielded distinct subtypes of melanomas, characterized by distinct prognoses, different transcriptional function profiles of DDR and immunity, and different responses to immunotherapy. A simplified 4‐CpG risk‐score signature was built and validated for clinical utility.

## 2 INTRODUCTION

Melanomas are among the most frequent and aggressive human skin cancers and are commonly associated with distant metastasis and poor prognosis.^1^ Conventional DNA‐damaging therapeutic strategies represent the mainstream adjuvant treatment for melanomas by inducing DNA damage and posing threats to the genomic integrity of tumor cells.^2^ In response, melanoma cells counteract and survive these genotoxic threats by fine‐tuning their DDR machinery, which involves complex networks of DNA damage sensors, mediators, and effectors.^3^, ^4^, ^5^, ^6^ In addition to determining the response to genotoxic treatments, tumor intrinsic DDR machinery has recently been highlighted as a central player in orchestrating the tumor immune microenvironment and determining the immunotherapy response.^7^ However, melanomas are characterized by high heterogeneity in tumor intrinsic DDR function and a high incidence of dysregulation and dysfunction in the DDR system.^3^, ^4^, ^5^, ^6^ Therefore, a comprehensive characterization of the DDR machinery may provide useful information on the prognosis, progression, and treatment response of melanomas.

DNA methylation is critical for gene expression control.^8^, ^9^, ^10^, ^11^ Like many human cancers, melanomas are commonly accompanied by genome‐wide DNA methylation alterations, resulting in altered transcriptomes that cover DDR genes.^12^ Several studies have highlighted the biological implications of epigenetic alterations in individual DDR genes or pathways in melanomas.^13^, ^14^, ^15^ However, there is still a lack of evidence evaluating the impacts of the global DDR DNA methylation landscape on melanoma biology and immunity in particular.

In this study, by incorporating a comprehensive genetic map of the human cell DDR system that was identified by a landmark clustered regularly interspaced short palindromic repeats (CRISPR)‐based screening study^16^ into a series of melanoma cohorts with genome‐wide DNA methylation microarray data, we revealed that global DNA methylation of DDR genes may correlate with survival, local immune status, and immunotherapy response in melanomas, which may not be contributed by different levels of tumor mutation burden (TMB). Hierarchical clustering analysis (HCA) utilizing different subsets of prognostically (and transcriptionally) relevant CpGs further yielded distinct melanoma subtypes featuring different prognoses, transcriptional function profiles of DDR and immunity, and immunotherapy responses. Finally, to simplify the DDR methylation‐based classification, we constructed and validated a 4‐CpG risk‐score signature to predict survival, local immune status, and immunotherapy response in melanomas.

## 3 METHODS

### 3.1 Melanoma public data sets

Genome‐wide DNA methylation data profiled by Illumina HumanMethylation 450k array, HTSeq‐transcriptome data in FPKM Format, and clinical information of 475 patients diagnosed with melanomas from The Cancer Genome Atlas (TCGA‐SKCM) were downloaded from UCSC Xena (https://xena.ucsc.edu/).^17^ Moreover, public melanoma cohorts were retrieved from the Gene Expression Omnibus (https://www.ncbi.nlm.nih.gov/geo) under accession numbers GSE144487,^18^ GSE51547,^19^ and GSE175699,^20^ respectively. Briefly, GSE144487^18^ included 196 melanoma samples profiled using the Illumina HumanMethylation EPIC array. GSE51547^19^ included 50 advanced melanoma (Stage III/IV) samples profiled using the Illumina HumanMethylation 450k array. Finally, GSE175699^20^ included 65 metastatic melanoma samples (Stage IV) profiled by the Illumina Human Methylation EPIC array, where information on response to immune checkpoint blockade (ICB) was available. Within the Illumina Human Methylation platform, the methylation signal of each CpG was summarized as a β value that provides a continuous and quantitative measurement of DNA methylation ranging from 0 (completely unmethylated) to 1 (completely methylated).^21^ The β value is mainly used for biological interpretation. In this study, the β value was transformed to the *M* value for statistical analysis, which has a logit transformation relationship with the β value.^21^ Missing data were imputed using Impute R package. Additional information on the molecular detection platform and data processing can be found in the original study.

### 3.2 Selection of DDR CpG methylation candidates

Initial CpG selection was performed by removing CpGs not interrogated on both the 450k and EPIC platforms, those targeting the sex chromosomes, those associated with single‐nucleotide polymorphisms, and those with invalid data. Batch effects across different data sets were adjusted using *M*‐value transformation^21^ and the Empirical Bayes method.^22^ In the present study, the human cell DDR machinery was defined as the genetic map of 842 core genes reported by a genome‐scale CRISPR/Cas9‐based screen study in human cells against 27 genotoxic agents.^16^ By intersecting the 842 DDR genes with the initially selected CpGs, 10,903 CpGs (located in genomic regions of 753 DDR genes) were left. A discovery–validation approach was employed for secondary CpG selection, with the TCGA‐SKCM setting as the discovery set. Sequential selection criteria were used in TCGA‐SKCM samples, including (1) CpGs with high variability in DNA methylation (standard deviation of β value > .2), (2) CpGs having a significant correlation with overall survival (OS), and (3) CpGs showing apparent and significant correlation with corresponding gene expression (absolute Pearson correlation coefficient value > 0.3 and *p* < .05).

### 3.3 Hierarchical clustering analysis (HCA)

HCA is an algorithm for grouping data points with similar properties.^23^ In this study, HCA was used to identify subsets of melanoma with similar DNA methylation patterns. The Euclidean distance and complete linkage methods were used for the HCA.

### 3.4 Risk‐score model construction and validation

After secondary CpG selection, 36 prognostically and transcriptionally relevant CpGs that were significantly associated with both OS and local gene expression remained. To further reduce data dimensionality and simplify the HCA‐based classification, the 36‐CpG panel was incorporated into a multivariable Cox regression model using a likelihood ratio test and a forward selection approach. Four CpGs that may independently and complementarily predict OS were identified and combined using a risk score model. For each sample, a risk score was calculated and equated to the sum of the *M*‐values of each CpG weighted by its multivariate Cox coefficient. The median risk score from TCGA‐SKCM was predefined as the cutoff for stratifying low‐risk and high‐risk tumors.

### 3.5 Gene set variation analysis (GSVA)

The gene set activity scores for each sample were calculated using *GSVA* R package (version 1.43.1).^24^ Gene sets (c5.go.bp.v2023.1 Hs symbols) were downloaded from The Molecular Signatures Database (www.gsea-msigdb.org/gsea/msigdb).^25^ The differences in the gene set activity scores for each sample were visualized using heatmaps.

### 3.6 Estimation of stromal and immune cells in malignant tumor tissues using expression data (ESTIMATE)

ESTIMATE can calculate the scores for tumor purity, level of stromal cells present, and infiltration level of immune cells in tumor bulks based on gene expression data.^26^ ESTIMATE scores were calculated for TCGA‐SKCM samples using *estimate* R package (version 1.0.13) based on RNA‐seq data in counts per million format**.**

### 3.7 Tumor‐infiltrating immune cells (TIIC) abundance and proportion

The abundance of 28 TIICs in bulk tumors was estimated using single‐sample gene set enrichment analysis (ssGSEA)^27^ over the 782‐gene signature reported by Charoentong et al.^28^ The abundance of the 28 TIICs in the tumor samples was summarized as normalized enrichment scores.^28^ CIBERSORT estimates the proportion of each immune cell type among 22 TIICs within a single sample.^29^ For TCGA‐SKCM, CIBERSORT data were downloaded from the TIMER2.0 database (http://timer.cistrome.org/).^30^

### 3.8 Tumor mutation burden (TMB) calculation

Simple nucleotide variation data from the “Masked Somatic Mutation” category in TCGA‐SKCM were downloaded using *TCGAbiolinks* R package (version 2.29.6). The R package “maftools” (version 2.14.0) was used to analyze the mutation annotation format file, and TMB was calculated by dividing the total number of nonsynonymous mutations by the total length of exons (38 Mb).

### 3.9 The tumor immune dysfunction and exclusion (TIDE)

The TIDE method was developed to predict the ICB response by simulating the mechanisms of tumor immune evasion (including T cell dysfunction and T cell exclusion).^31^ TIDE was run for TCGA‐SKCM samples using online software (http://tide.dfci.harvard.edu).^31^

### 3.10 Statistical analysis

Frequency data were tested using Fisher's exact test or Chi‐square test. Continuous data were tested using Student's *t*‐test or Mann–Whitney test. Survival data were estimated using the Kaplan–Meier method and compared using the log‐rank test. The correlation between DNA methylation and gene expression data was evaluated using Pearson correlation analysis. Prognostic correlations and independence were evaluated using univariate and multivariate Cox regression models. Meta‐analysis was conducted where the inverse‐variance approach was applied using either fixed‐ or random‐effect models based on the heterogeneity test, with *p* < .1 or *I* ^2^ > 50% as statistical significance. The prognostic performance was also assessed by the receiver operating characteristic (ROC) curve and the area under the curve (AUC). All calculations were performed using SPSS statistics (SPSS software Inc.) and R software, with two‐sided *P* values ≤ .05, for statistical significance.

## 4 RESULTS

### 4.1 Global DNA methylation variations of DDR correlate with distinct prognosis and local immune status in melanomas

**Figure 1:**
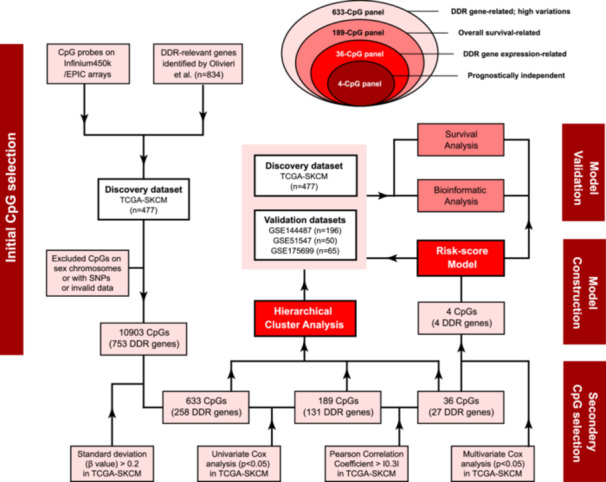
The study workflow for the CpG selection, model construction, and validation based on DNA methylation landscape of DNA damage response (DDR) genes for melanomas.

**Figure 2:**
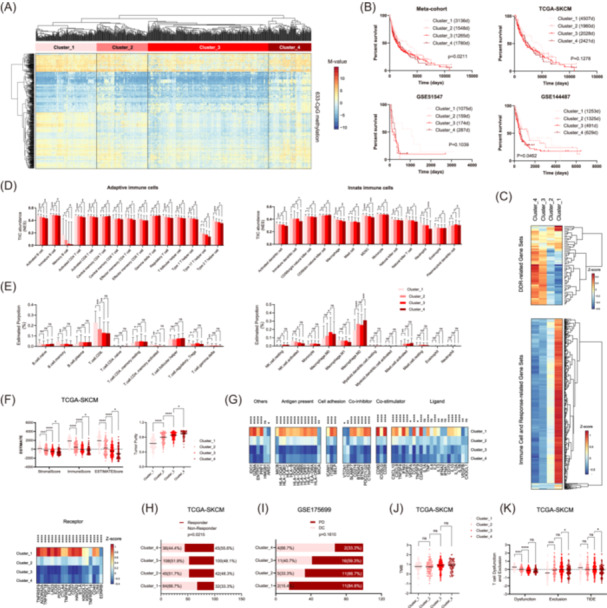
Hierarchical clustering analysis (HCA) utilizing the 633 DNA damage response (DDR) relevant CpGs identified four prognostically and biologically distinct melanoma clusters. (A) Heat map of the hierarchical clusters defined by DNA methylation of 633 DDR‐relevant CpGs among all melanoma cohorts (TCGA‐SKCM, GSE175699, GSE144487, and GSE51547 collectively). Each row represents a CpG and each column represents a sample that is grouped by HCA. Clinical features are also indicated for each sample. (B) Survival comparison among the four clusters in each cohort; (C) Gene set variation analysis enrichment levels of DDR and immune cell and response‐relevant biological processes among the four clusters; (D) The abundance of tumor‐infiltrating immune cells (TIICs), including adaptive immune cells and innate immune cells, in tumor bulks from TCGA‐SKCM, estimated by single‐sample gene set enrichment analysis; (E) The relative proportion of TIICs, including adaptive immune cells and innate immune cells, in each tumor bulk from TCGA‐SKCM, estimated by CIBERSORT; (F) Tumor stromal and immune scores and tumor purity estimated by estimation of stromal and immune cells in malignant tumor tissues using expression (ESTIMATE) method; (G) The expression levels of immune checkpoint molecules among the four clusters from TCGA‐SKCM; (H) Responder and nonresponder distribution among the four clusters from TCGA‐SKCM, predicted by tumor immune dysfunction and exclusion (TIDE); (I) Distribution of patients with progressive disease (PD) or disease control (DC) after immunotherapy among the four clusters from GSE175699; (J) Comparison of tumor mutation burden (TMB) among the four clusters from TCGA‐SKCM; (K) Comparison of T cell dysfunction and exclusion among the four clusters from TCGA‐SKCM; statistical significance was indicated at the level of ns > 0.05, *<0.05, **<0.01, ***<0.001, and ****<0.0001. ns, nonsignificant.

After initial and secondary CpG selection, 633 DDR‐relevant CpGs were identified as highly variable methylation sites in melanomas (Figure 1 and Table S1). HCA on the 633‐CpG panel classified TCGA‐SKCM samples into four tumor clusters (Figure 2A). In TCGA‐SKCM, the clusters were not apparently associated with age, tumor stage (early vs. advanced), metastasis, ulceration, thickness (<2 mm vs. ≥2 mm), and pre‐/post‐surgery treatments (data not shown). Among the clusters, OS differed with marginal statistical significance, and Cluster_1 showed the best OS (Figure 2B). GSVA showed that the four clusters were associated with different transcriptional profiles of DDR and immune‐relevant gene sets (Figure 2C). Briefly, DDR gene sets were highly enriched in Clusters _3 and _4, whereas immunity‐relevant gene sets were enriched in Cluster_1 (Figure 2C). ssGSEA showed that most of the 28 TIICs were differentially distributed across the clusters, with Cluster_1 harboring the most abundant TIIC infiltrations in tumor bulks (Figure 2D). CIBERSORT showed that the relative proportions of some immune cell types in all TIICs were different across the clusters; specifically, the relative proportions of antitumor immune cells, such as CD8 + T cells and M1 macrophages, were highest in Cluster_1 (Figure 2E). In line with the above bioinformatic analyses, ESTIMATE showed that Cluster_1 was also associated with the highest levels of ImmuneScore, StromalScore, and ESTIMATEScore and, accordingly, the lowest level of tumor purity (Figure 2F). A comparison of immune checkpoint molecules^32^ showed that most of these molecules were highly expressed in Cluster_1 (Figure 2G). Regarding the response to immunotherapy, TIDE showed that despite having the most active local immune functional profiles and the highest abundance of TIICs, Cluster_1 appeared to be less sensitive to ICB therapy (Figure 2H). By correlating the HCA‐based clusters with therapeutic outcomes (progressive disease [PD] vs. disease control) after ICB therapy in GSE175699, we again confirmed ICB resistance in Cluster_1 (Figure 2I). Further analysis revealed that the four clusters were not associated with different TMB values (Figure 2J), a candidate parameter reflecting tumor immunogenicity and immunotherapy efficacy,^33^ but were characterized by different T cell‐mediated immune evasion modes (Figure 2K). Specifically, Cluster_1 showed the highest level of T cell dysfunction but the lowest level of T cell exclusion (Figure 2K). These data indicate that severe T cell dysfunction may contribute to the ICB resistance observed in cluster 1.

### 4.2 DNA methylation patterns of 189 prognostically relevant CpGs define three melanoma subtypes characteristic of distinct prognosis and transcriptional profiles of DDR and immunity

**Figure 3:**
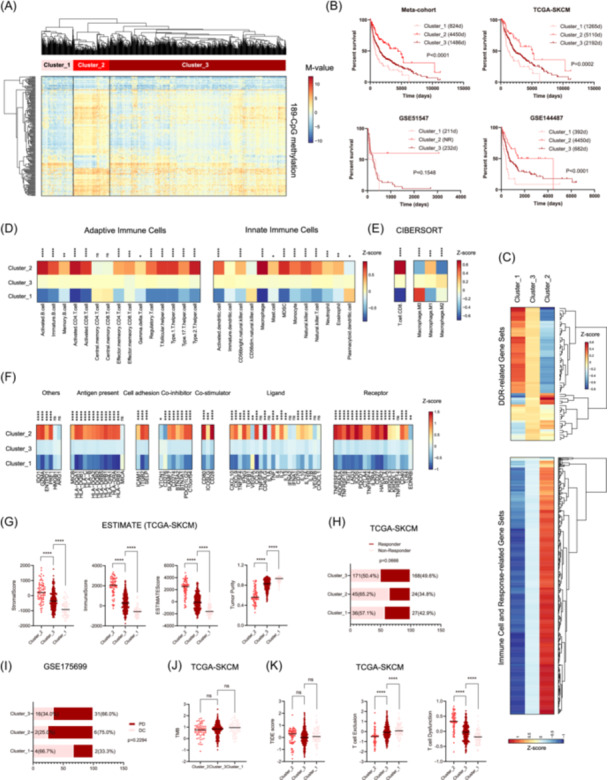
Hierarchical clustering analysis (HCA) utilizing the 189 DNA damage response (DDR) relevant CpGs identified three prognostically and biologically distinct melanoma clusters. (A) Heat map of the hierarchical clusters defined by DNA methylation of 189 DDR‐relevant prognostic CpGs among all melanoma cohorts (TCGA‐SKCM, GSE175699, GSE144487, and GSE51547 collectively). Each row represents a CpG and each column represents a sample that is grouped by HCA. Clinical features are also indicated for each sample. (B) Survival comparison among the three clusters in each cohort; (C) Gene set variation analysis enrichment levels of DDR and immune cell and response‐relevant biological processes among the three clusters; (D) The abundance of tumor‐infiltrating immune cells, including adaptive immune cells and innate immune cells, in tumor bulks from TCGA‐SKCM, estimated by single‐sample gene set enrichment analysis; (E) The relative proportion of CD8 + T cells and macrophage subtypes (e.g., M0, M1, M2) in each tumor bulk from TCGA‐SKCM, estimated by CIBERSORT; (F) The expression levels of immune checkpoint molecules among the three clusters from TCGA‐SKCM; (G) Tumor stromal and immune scores and tumor purity estimated by estimation of stromal and immune cells in malignant tumor tissues using expression (ESTIMATE); (H) Responder and nonresponder distribution among the three clusters from TCGA‐SKCM, predicted by tumor immune dysfunction and exclusion (TIDE); (I) Distribution of patients with progressive disease (PD) or disease control (DC) after immunotherapy among the three clusters from GSE175699; (J) Comparison of tumor mutation burden (TMB) among the four clusters from TCGA‐SKCM; (K) Comparison of T cell dysfunction and exclusion among the three clusters from TCGA‐SKCM; statistical significance was indicated at the level of ns > 0.05, *<0.05, **<0.01, ***<0.001, and ****<0.0001. ns, nonsignificant.

To optimize the DDR methylation‐based classification, we further selected 189 prognostically relevant CpGs that were significantly correlated with OS in TCGA‐SKCM for HCA. Similarly, HCA in the 189‐CpG panel classified all samples into three tumor clusters (Figure 3A). In TCGA‐SKCM, the clusters were not significantly associated with age, tumor stage (early vs. advanced), metastasis, ulceration, thickness (<2 mm vs. ≥2 mm), and pre‐/post‐surgery treatments (data not shown). Among the three clusters, the OS was significantly different (Figure 3B). Cluster_2 showed the longest OS, followed by Cluster_3, and Cluster_1 showed the worst OS (Figure 3B). Bioinformatics analyses showed that Cluster_2 with the best prognosis was also associated with the highest enrichment of immunity‐relevant gene sets (Figure 3C), the highest abundance of TIICs (Figure 3D,E), the highest expression levels of immune checkpoint molecules (Figure 3F), and the highest levels of stromal score, immune score, and ESTIMATEScore (Figure 3G). In contrast, the DDR gene sets were the most repressed in Cluster_2 (Figure 3C). Regarding immunotherapy efficacy, TIDE predicted that Cluster_2 might be associated with a higher proportion of nonresponders to ICB therapy in TCGA‐SKCM (Figure 3H), which was also observed in GSE175699 (Figure 3I). Similarly, further analysis showed that the three clusters were not associated with different TMB (Figure 3J) but featured different T cell‐mediated immune evasion modes (Figure 3K). Specifically, Cluster_2 may mainly suffer from T cell dysfunction, whereas Cluster_1 may mainly suffer from the exclusion of T cells (Figure 3K).

### 4.3 DNA methylation patterns of 36 prognostically and transcriptionally relevant CpGs still define two melanoma subtypes with distinct prognosis and transcriptional profiles of DDR and immunity

**Figure 4:**
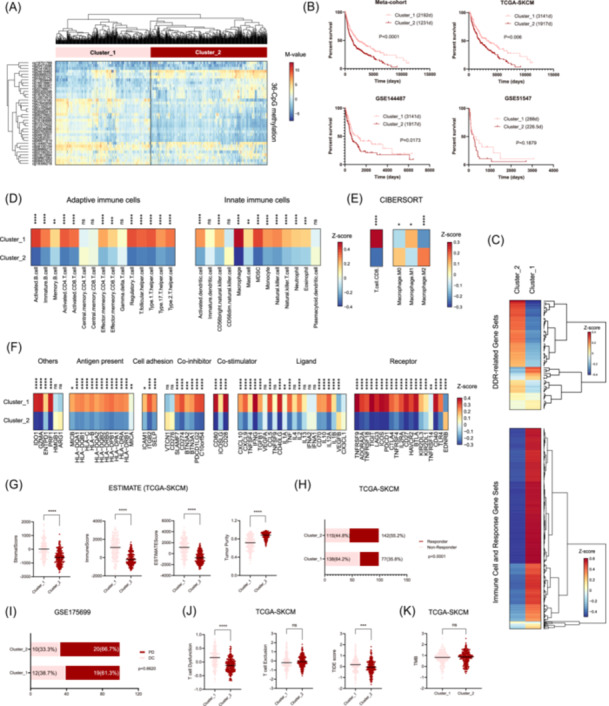
Hierarchical clustering analysis (HCA) utilizing the 36 DNA damage response (DDR) relevant CpGs identified two prognostically and biologically distinct melanoma clusters. (A) Heat map of the hierarchical clusters defined by DNA methylation of 36 prognostically and transcriptionally relevant CpGs among all melanoma cohorts (TCGA‐SKCM, GSE175699, GSE144487, and GSE51547 collectively). Each row represents a CpG and each column represents a sample that is grouped by HCA. Clinical features are also indicated for each sample. (B) Survival comparison among the two clusters in each cohort; (C) Gene set variation analysis enrichment levels of DDR and immune cell and response‐relevant biological processes between the two clusters; (D) The abundance of tumor‐infiltrating immune cells, including adaptive immune cells and innate immune cells, in tumor bulks from TCGA‐SKCM, estimated by single‐sample gene set enrichment analysis; (E) CD8 + T cells and macrophage subtypes (e.g., M0, M1, M2) in each tumor bulk from TCGA‐SKCM, estimated by CIBERSORT; (F) The expression levels of immune checkpoint molecules between the two clusters from TCGA‐SKCM; (G) Tumor stromal and immune scores and tumor purity estimated by estimation of stromal and immune cells in malignant tumor tissues using expression (ESTIMATE) method; (H) Responder and nonresponder distribution between the two clusters from TCGA‐SKCM, predicted by tumor immune dysfunction and exclusion (TIDE); (I) Distribution of patients with progressive disease (PD) or disease control (DC) after immunotherapy between the two clusters from GSE175699; (J) Comparison of T cell dysfunction and exclusion between the two clusters from TCGA‐SKCM; (K) Comparison of tumor mutation burden (TMB) between the two clusters from TCGA‐SKCM; statistical significance was indicated at the level of ns > 0.05, *<0.05, **<0.01, ***<0.001, and ****<0.0001. ns, nonsignificant.

DNA methylation plays a key role in controlling gene transcription control.^34^ Among the 189‐CpG panel, 36 CpGs were found to be closely correlated with the expression of DDR genes individually harboring each CpG (Tables 1 and S1). HCA of these prognostically and transcriptionally relevant CpGs also yielded two distinct tumor clusters (Figure 4A). In line with the above findings, the clusters were not apparently associated with age, tumor stage (early vs. advanced), ulceration, thickness (<2 mm vs. ≥2 mm), and pre‐/post‐surgery treatments, except for a higher proportion of primary tumors in Cluster_2 in TCGA‐SKCM (data not shown). The defined Cluster_1 with favorable OS (Figure 4B) had a more active local immune response, characterized by high enrichment of immunity‐relevant gene sets (Figure 4C), high abundance of TIICs (Figure 4D,E), high expression of immune checkpoint molecules (Figure 4F), and high stromal score, immune score, and ESTIMATEScore (Figure 4G). Similarly, DDR gene sets were mostly downregulated in Cluster_1 (Figure 4C). Regarding the immunotherapeutic response, TIDE predicted that Cluster_1 would be more resistant to ICB therapy than Cluster_2 (Figure 4H), but this was not observed in GSE175699 (Figure 4I). In TCGA‐SKCM, Cluster_1 was found to be associated with more severe dysfunction of T cells (Figure 4J), while the T cell exclusion score and TMB were not significantly different between the two clusters (Figure 4J,K).

**Table 1:** Characteristics of the 36 prognostically and biologically relevant CpGs.

Illumina probe	Gene symbol	Relation to CpG island^a^	Relation to gene	Pearson *r* coefficient^b^	Univariate Cox regression coefficient^c^	Risk‐score signature
cg25473596	AGK	Shore	TSS1500	−0.344	0.151	Yes
cg01812894	ALDH1A1	Open sea	Body	−0.646	0.102	
cg00300298	BCL2L1	Shore	Body	−0.507	0.076	
cg12873919	BCL2L1	Shore	3′UTR	−0.441	0.122	Yes
cg18787420	BCL2L1	Shore	Body	−0.456	0.123	
cg04819351	C15orf41	Shore	5′UTR	−0.348	−0.101	
cg06871919	C5orf22	Open sea	Body	−0.306	−0.151	
cg21410633	CAP1	Open sea	TSS1500	−0.317	0.081	
cg23235142	CCM2	Open sea	TSS200	−0.350	0.129	
cg01574233	CDK2	Shore	1stExon	−0.564	−0.167	
cg21074190	CDK2	Shore	TSS1500	−0.693	−0.150	
cg12377256	CDK2	Shore	Body	−0.470	−0.138	
cg16190688	CDK2	Shore	TSS1500	−0.563	−0.135	
cg24816460	CDYL	Island	TSS200	0.360	0.087	
cg18399218	CLCN3	Open sea	TSS1500	−0.326	−0.148	
cg27645858	COPS8	Open sea	TSS1500	−0.378	−0.198	
cg03752885	DAPK3	Shore	Body	−0.375	−0.215	
cg26585416	DAPK3	Shore	TSS1500	−0.339	−0.206	Yes
cg14485744	EXT1	Open sea	Body	−0.319	−0.116	Yes
cg23434592	FAM135B	Open sea	TSS200	0.477	−0.117	
cg06276653	FAM135B	Open sea	TSS200	0.454	−0.115	
cg20799917	GALM	Open sea	5′UTR	−0.613	0.084	
cg08100159	ITGB1BP1	Shelf	3′UTR	−0.403	−0.145	
cg19751789	LDLR	Shore	1stExon	−0.692	0.058	
cg27182012	LMNA	Open sea	Body	−0.451	−0.108	
cg16518861	NFKBIA	Shore	3′UTR	−0.531	0.091	
cg00689225	NFKBIA	Shore	TSS1500	−0.597	0.117	
cg00762605	NHEJ1	Shelf	3′UTR	−0.328	−0.134	
cg20036996	PUF60	Open sea	TSS1500	−0.432	−0.103	
cg09861583	RAMP1	Shore	5′UTR	−0.324	−0.077	
cg08457620	RTN4RL1	Shore	TSS200	−0.562	−0.079	
cg19619387	RTN4RL1	Island	Body	0.305	0.094	
cg17859448	S1PR1	Shore	TSS200	−0.406	0.119	
cg25835351	SEPHS1	Open sea	TSS200	−0.308	−0.141	
cg02190353	SLC25A37	Open sea	Body	−0.347	−0.070	
cg08989214	ZCCHC14	Open sea	TSS200	−0.331	−0.117	

^a^ Each CpG was classified into island, shore (1 to 2000 bp from island), shelves (2001 to 4000 bp from island), and open sea (>4000 bp from island) according to the distance to relevant CpG island regions.

^b^ Pearson's coefficient was calculated using paired DNA methylation and gene expression data from TCGA‐SKCM.

^c^ Univariate Cox regression coefficient was calculated using survival data from TCGA‐SKCM.

### 4.4 Construction and validation of a risk‐score signature of 4 DDR CpGs for predicting survival, local immune status, and immunotherapy response in melanomas

**Figure 5:**
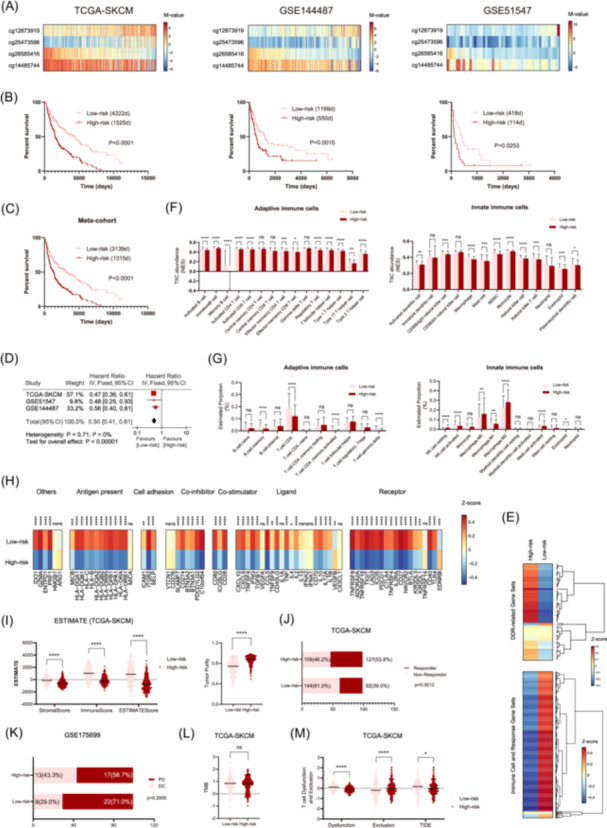
The prognostic and biological evaluation of a 4‐CpG risk score signature for melanomas. (A) Heat maps of the 4‐CpG methylation in each cohort. Each row represents a CpG and each column represents a sample that is ranked by assigned risk scores; Clinical and molecular features are also indicated for each sample. (B) Prognostic performance of the 4‐CpG risk score signature in each cohort; low‐risk and high‐risk groups were stratified by the median risk score value (−0.3369) from TCGA‐SKCM; (C) Pooled survival comparison between low‐risk and high‐risk tumors at individual patient level; (D) Meta‐analysis of the low‐risk and high‐risk groups at cohort level; (E) Gene set variation analysis enrichment levels of DNA damage response and immunity‐relevant biological processes; (F) The abundance of tumor‐infiltrating immune cells (TIICs), including adaptive immune cells and innate immune cells, in tumor bulks from TCGA‐SKCM, estimated by single‐sample gene set enrichment analysis; (G) The relative proportion of TIICs, including adaptive immune cells and innate immune cells, in each tumor bulk from TCGA‐SKCM, estimated by CIBERSORT; (H) The expression levels of immune checkpoint molecules between low‐risk and high‐risk tumors from TCGA‐SKCM; (I) Tumor stromal and immune scores and tumor purity estimated by estimation of stromal and immune cells in malignant tumor tissues using expression (ESTIMATE); (J) Responder and nonresponder distribution between the low‐risk and high‐risk tumors from TCGA‐SKCM; (K) Distribution of patients with progressive disease (PD) or disease control (DC) after immunotherapy between the risk subgroups from GSE175699; (L) Comparison of tumor mutation burden (TMB) between the risk subgroups from TCGA‐SKCM; (M) Comparison of T cell dysfunction and exclusion among the three clusters from TCGA‐SKCM; statistical significance was indicated at the level of ns > 0.05, *<0.05, **<0.01, ***<0.001, and ****<0.0001. ns, nonsignificant.

Inspired by the above classifications, defined by different combinations of DDR DNA methylation alterations, we aimed to simplify these HCA‐based models for clinical utility. By employing a multivariable Cox regression method and a risk‐score algorithm (Figure 1), we identified four prognostically and transcriptionally relevant CpGs (Table 1 and Figure 5A), each of which provides independent and complementary predictive information for patient survival. Using the median risk score as the cutoff (−0.3369 from TCGA‐SKCM), all patients were divided into low‐risk and high‐risk groups (Figure 5B). In TCGA‐SKCM, the low‐risk group was significantly associated with older age (*p* = .0044), more primary cases (*p* = .0004), and more ulcerations (*p* = .0371). The pre‐/post‐surgery treatments were similar between the low‐ and high‐risk groups (data not shown). The prognostic performance of the 4‐CpG risk score signature in stratified cohorts by the clinical variables was shown in Figure S1. In both the discovery set (TCGA‐SKCM) and validation sets (GSE144487 and GSE51547 collectively), low‐risk patients were significantly associated with longer OS than high‐risk patients (Figure 5B–D). Multivariable Cox regression analyses revealed prognostic independence of the 4‐CpG risk score signature in patients with melanoma (Table S2). The prognostic performance of the 4‐CpG signature was also evaluated by AUC‐ROC at indicated times (Figure S2). Bioinformatics analyses showed that low‐risk tumors were associated with repressed transcriptional profiles of DDR, activated transcriptional profiles of immunity (Figure 5E), abundant infiltration of TIICs (Figure 5F,G), upregulated expression of immune checkpoint molecules (Figure 5H), and high levels of stromal, immune, and ESTIMATEScore (Figure 5I). Regarding immunotherapy efficacy, both the predicted nonresponders in TCGA‐SKCM and actual patients with PD after ICB therapy in GSE175699 were more frequent in the low‐risk groups (Figure 5J,K). Finally, low‐risk tumors were associated with severe T cell dysfunction and similar TMB (Figure 5L,M).

## 5 DISCUSSION

Comprehensive studies have proposed that the DDR machinery of tumor cells may have profound effects on the tumor immune microenvironment.^3^, ^4^, ^5^, ^6^ Dysregulation and dysfunction of the tumor intrinsic DDR system may consequently determine the local tumor immunophenotype and efficacy of immunotherapy and ICB therapy in particular.^3^, ^4^, ^5^, ^6^ Alterations in DNA methylation represent critical driver events that cause deficiencies in individual DDR genes or pathways in tumor cells.^8^, ^9^, ^10^, ^11^ However, evidence evaluating the effects of the global DNA methylation landscape of the human cell DDR system on melanoma immunity is lacking.

In the present study, we first showed that global DNA methylation variations in DDR genes are associated with distinct prognosis, transcriptional profiles of DDR, and local immunity in melanomas. Specifically, HCA utilizing a total of 633 CpGs that were located within genomic regions of DDR genes identified a distinct melanoma subtype (Cluster_1), characteristic of low expression of DDR‐related genes, high expression of immune cell‐ and immune response‐related genes, and abundant immune cell infiltration in tumors. Previous studies have indicated that immune cell infiltration (particularly T cell infiltration), M1 macrophage polarization, and high tumor antigen expression (e.g., HLA) are correlated with favorable prognosis in melanomas.^35^ In addition, reduced DDR functional profiles may contribute to the favorable prognosis of this tumor subset via enhancing tumor cell antigenicity, inducing immunogenic cell death, and triggering tumor cell‐autonomous immune responses.^36^ However, the immunologically active Cluster_1 seemed to show less sensitivity to immunotherapy, as indicated by the bioinformatics analysis from TIDE and clinical data from GSE175699. The tumor microenvironments in Cluster_1 can be intricate and conflicting. When tumors encounter more abundant infiltrations of immune cells, they may have a greater chance to develop heightened immune escape mechanisms amidst active antitumor immune responses, including increased expression of immune checkpoint molecules, enrichment of regulatory T cells, M2 macrophages, and myeloid‐derived suppressor cells, and increased T cell dysfunction in the tumor microenvironment, as observed in our study (Figure 2). This assumption was also supported by the close correlation between the bioinformatically calculated immune cell infiltration levels and T cell dysfunction (data not shown). Previous studies have indicated that all these characteristics played significant roles in resistance to immunotherapy.^37^ TMB refers to the frequency of mutations in the coding regions of somatic cells and may reflect the antigenicity of tumor cells and the likelihood of being recognized by the immune system.^38^ In our study, somatic mutation analysis revealed similar TMB values across the four clusters, suggesting that TMB may not contribute to distinct immunotherapy responses among the clusters. In summary, the above findings preliminarily describe the relationship between epigenetic abnormalities of the DDR system and melanoma immunity.

The 633‐CpG HCA model was not considered an optimal melanoma classification scheme because the other clusters (Clusters 2 to 4) showed limited differences in OS and local immunogenomic profiles. To optimize melanoma classification, we selected CpGs that were significantly correlated with OS in TCGA‐SKCM to run HCA. Clustering utilizing 189 prognostically relevant CpGs yielded three distinct subtypes with apparent differences in OS and transcriptional function profiles of immunity and DDR. Among the three clusters, Cluster_2 was characterized by the longest OS, the most repressed transcriptional profiles of DDR gene sets, and the most active local immune response, including activated immune transcriptional profiles, abundant infiltration of TIICs, and upregulation of immune checkpoint molecules. In contrast, Cluster_3, which had the poorest prognosis, was characterized by a highly enriched transcriptional profile of DDR gene sets and an inactive local immune microenvironment. In addition, Cluster_1, with an intermediate prognosis, showed an intermediate DDR and immune function status. The three clusters were also found to exhibit different responses to ICB therapy, with the immunologically most active Cluster_2 having the worst response. In summary, characterization of 189‐CpG‐based HCA clusters may provide a clear example illustrating the relationships among DNA methylation, DDR function, local tumor immunity, immunotherapy efficacy, and survival in melanomas. It is widely known that tumor‐specific DNA methylation affects tumor behavior, mainly by regulating gene expression.^8^ Therefore, from the 189 CpGs, we further selected 36 CpGs that were closely correlated with local gene expression to run HCA. Similarly, the HCA model utilizing the 36 prognostically and transcriptionally relevant CpGs yielded two distinct melanoma subtypes with distinct survival and transcriptional function profiles of DDR and immunity and similar TMB status. Taken together, the above findings highlight that DNA methylation of the DDR system may have critical roles in determining the local tumor immune microenvironment and consequently the prognosis and immunotherapy efficacy for melanomas, which is likely to be independent of TMB.

Despite the encouraging findings revealed by the HCA‐based clusters, these models had limited value for clinical utility because they were not convenient for classifying newly tested individual patients. Therefore, we built a clinically applicable model based on DDR DNA methylation. A risk‐score signature employing four CpGs that can independently and complementarily predict the survival of patients with melanoma was constructed. Successful validation of the 4‐CpG signature in independent patient cohorts supported its prognostic robustness and independence in melanomas. Bioinformatic analyses revealed that the 4‐CpG signature was tightly correlated with distinct transcriptional function profiles of DDR and immunity, especially with distinct responses to ICB therapy. A literature review may provide biological and molecular evidence for the 4‐CpG signature. The signature comprised four CpGs that may affect the expression of four DDR genes: *AGK*, *BCL2L1*, *DAPK3*, and *EXT1*. Specifically, *AGK* encodes acylglycerol kinase, a mitochondrial membrane protein involved in lipid and glycerolipid metabolism.^39^ *AGK* has been widely reported to act as an oncogene in various cancers by regulating metabolism and mitochondrial function.^39^ A recent study also highlighted the critical role of *AGK* in establishing and maintaining CD8 + T‐cell metabolic and functional fitness.^40^ *BCL2L1* mRNA can be alternatively spliced to produce two isoforms: the antiapoptotic long‐form Bcl‐xL and the proapoptotic short‐form Bcl‐xS.^41^ Therefore, the splicing control of *BCL2L1* may play a central role in regulating the apoptotic process and therapeutic response in cancers.^41^ *DAPK3* encodes a death‐associated protein kinase that may play a role in a wide range of cellular processes including extracellular matrix separation, apoptosis, and autophagic vesicle formation.^42^ Previous studies have shown that *DAPK3* is commonly methylated in cancers, resulting in loss of its tumor suppressor function.^43^ A recent study reported that *DAPK3* might drive tumor‐intrinsic immunity through the cGAS‐STING‐INF‐β pathway, which has been increasingly highlighted as a central interface between the cellular DDR system and tumor immunity.^44^ *EXT1* encodes an endoplasmic reticulum‐resident type II transmembrane glycosyltransferase that is involved in heparan sulfate biosynthesis. Similar to *DAPK3*, *EXT1* is commonly associated with hypermethylated CpG islands, reduced heparan sulfate production, and tumor suppressor‐like functions in various cancers.^45^ In melanomas, *EXT1* loss may promote metastatic spread through heparan sulfate deficiency^10^ and induce resistance to DNA‐damaging agents through intracellular signaling activation (e.g., EGFR/JNK/MEK/ERK signaling).^45^ In summary, emerging data from the literature have provided initial evidence supporting the implications of the 4‐CpG signature in melanoma biology.

The impact of DDR alterations on the local immune response has been increasingly documented in cancers.^3^, ^4^, ^5^, ^6^ Current evidence has proposed several approaches by which intrinsic DDR deficiency manipulated tumor immunophenotypes, including activation of immune response through cytosolic DNA sensing pathways or cell death signals, enhancement of tumor immunogenicity by increasing tumor mutations and genome instability, and reshaping local tumor immune microenvironments by regulating key immune checkpoint molecules (e.g., PD‐L1).^3^ In our DDR DNA methylation‐based clusters, we found that a highly activated DDR transcriptome is usually associated with decreased infiltration of TIICs and a repressed immune‐relevant transcriptional profile, including immune checkpoint molecules. However, the TMB of tumor bulks did not differ across the clusters, suggesting that DNA methylation of DDR may not affect tumor immunogenicity. Taken together, these findings indicate that DNA methylation may determine local immune microenvironments by affecting the transcriptional profiles of the DDR system, during which TMB‐induced alterations in tumor immunogenicity may have limited roles. The establishment of DDR methylation‐based immune‐relevant subtypes of melanomas may hold great significance in guiding the current treatment of melanomas. DDR methylation‐based classification may provide useful information for identifying subpopulations of patients who will most likely benefit from current immunotherapy. Moreover, DDR epigenetic classification may inspire the utility of epigenetic therapy for melanomas, encourage its combination with DNA‐damaging agents, DDR inhibitors, and immunotherapy, and provide useful guidance for their combinatorial use.

This study has several limitations. Genomic coverage was killed by limiting CpGs to those interrogated on both 450k and EPIC arrays to ensure a larger sample size. The correlation of our DDR methylation‐based classifications and functional status of tumor DDR and immunity has only been validated in TCGA‐SKCM owing to the lack of additional melanoma cohorts with multi‐omics molecular data. Clinical information and treatment modalities in particular were incomplete and could confound the final findings. The risk‐score model using microarray data is not ready for routine clinical use, and validation by alternative methods, such as pyrosequencing, is warranted for clinical utility. Finally, some of our findings are based on bioinformatic predictions, and the biological implications of DDR DNA methylation alterations have not yet been confirmed through experimentation.

## 6 CONCLUSIONS

In summary, the present study proposes a series of DDR DNA methylation‐based subtypes of melanomas with distinct prognoses, transcriptional function profiles of DDR and immunity, and immunotherapy response (Figure S3). These novel epigenetic DDR classification schemes for melanomas not only provide ideal models to illustrate the interrelationship among DNA methylation, DDR function, local tumor immunity, immunotherapy efficacy, and cancer prognosis but also provide useful information for predicting immunotherapy response and guiding the combinatorial use of epigenetic therapy, DDR inhibitors, DNA‐damaging agents, and immunotherapy.

Future studies are warranted to validate the clinical and molecular features of DDR DNA methylation‐based classification, modify the microarray‐based prognostic model, and explore the biological implications of epigenetically controlled DDR genes in melanoma.

## AUTHOR CONTRIBUTIONS

Min Wang, Xiao‐dong Zhang, and An‐an Yin contributed to the study conception and design. Min Wang, Xiao‐dong Zhang, Han‐qing Yang, and Yang Li performed material preparation, data collection, and analysis. Wen‐mei Chen and An‐an Yin wrote the first draft of the manuscript. All authors have read and approved the final manuscript.

## CONFLICT OF INTEREST STATEMENT

The authors declare no conflict of interest.

## ETHICS STATEMENT

The ethics statement is not applicable to this article.

## Supporting information

Supporting information.

Supporting information.

Supporting information.

Supporting information.

Supporting information.

Supporting information.

## Data Availability

The data sets used and/or analyzed during the current study are available from public databases, the UCSC Xena (http://xena.ucsc.edu/), and the Gene Expression Omnibus: https://www.ncbi.nlm.nih.gov/geo/).
