# Supplementary figures and images for: DNA methylation variations of DNA damage response correlate survival and local immune status in melanomas
Source: Immun Inflamm Dis. 2024 Sep 10;12(9):e1331. doi: 10.1002/iid3.1331 (PMC11386344; doi:10.1002/iid3.1331)

A

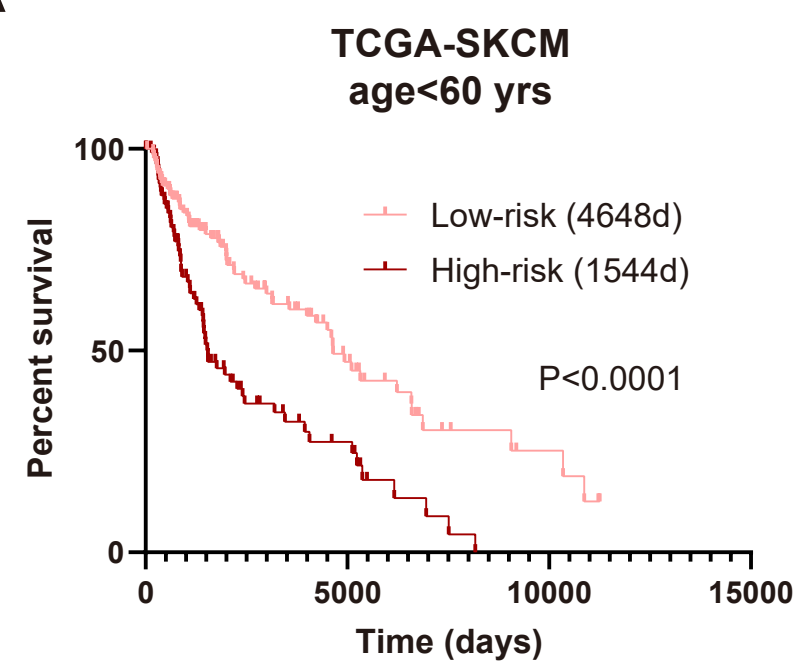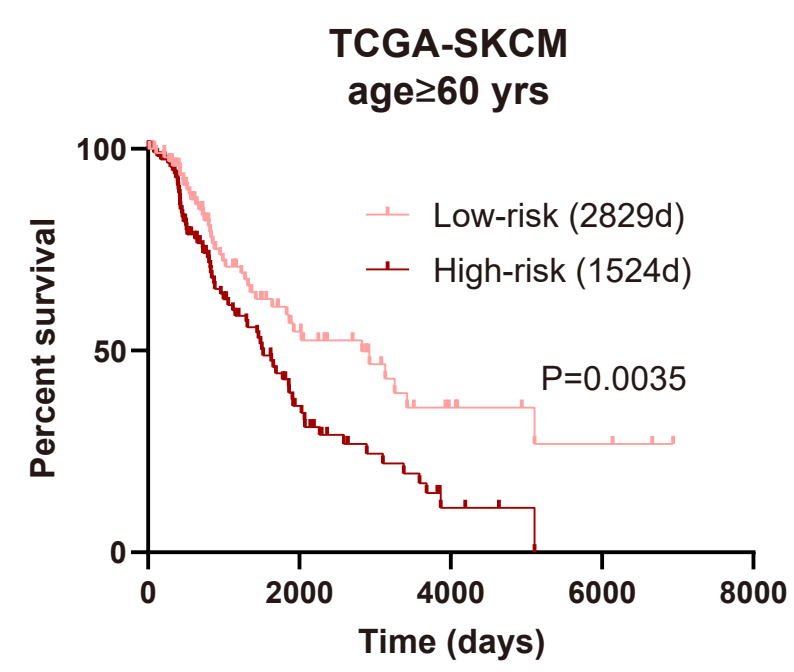

B

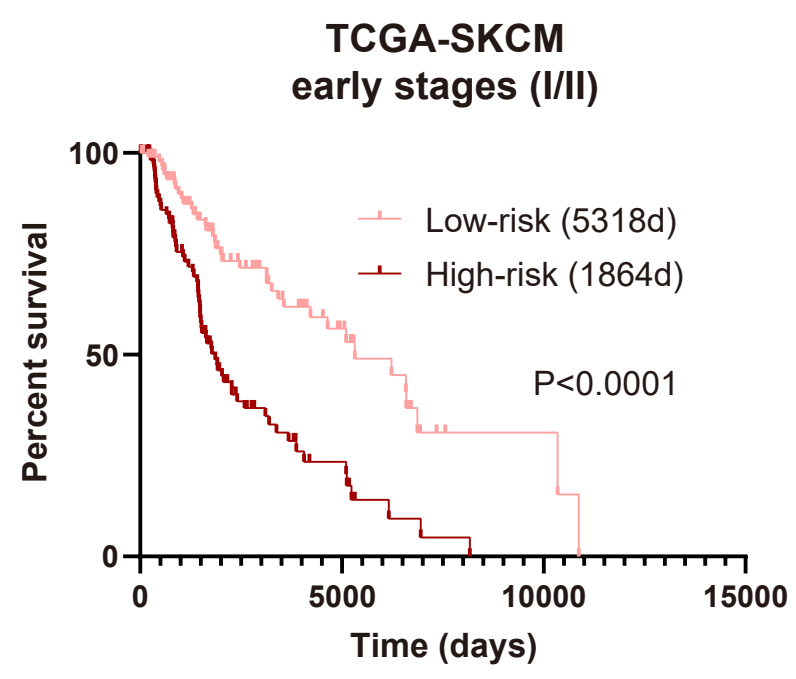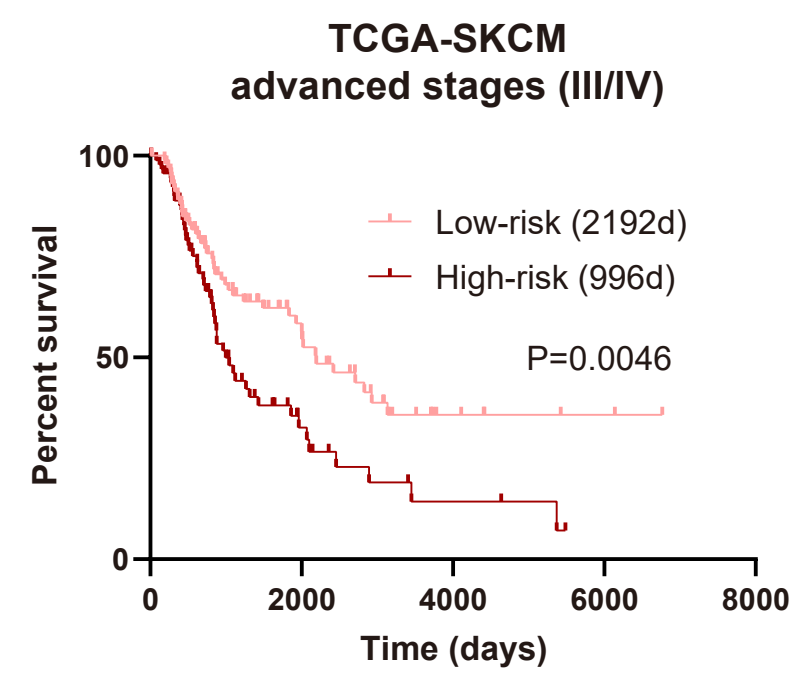

C

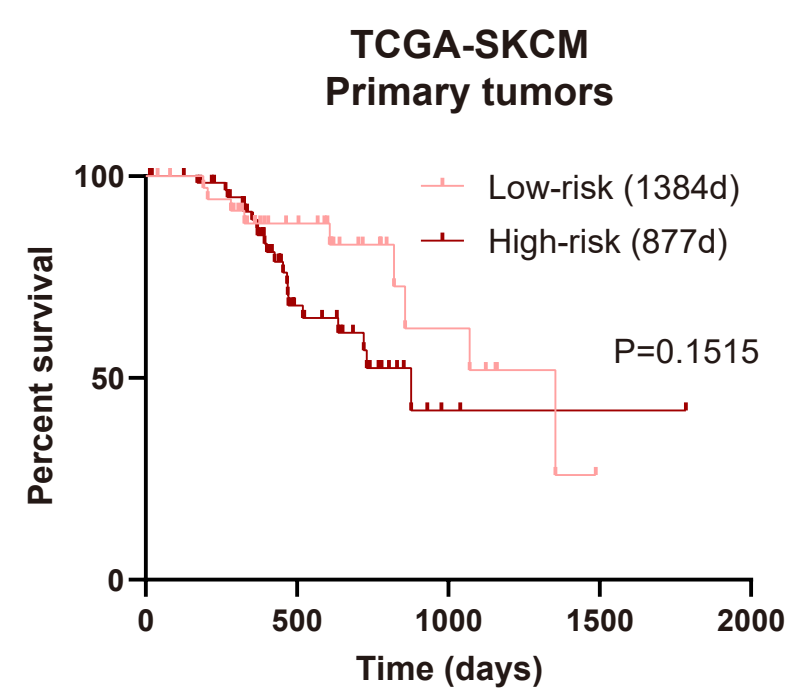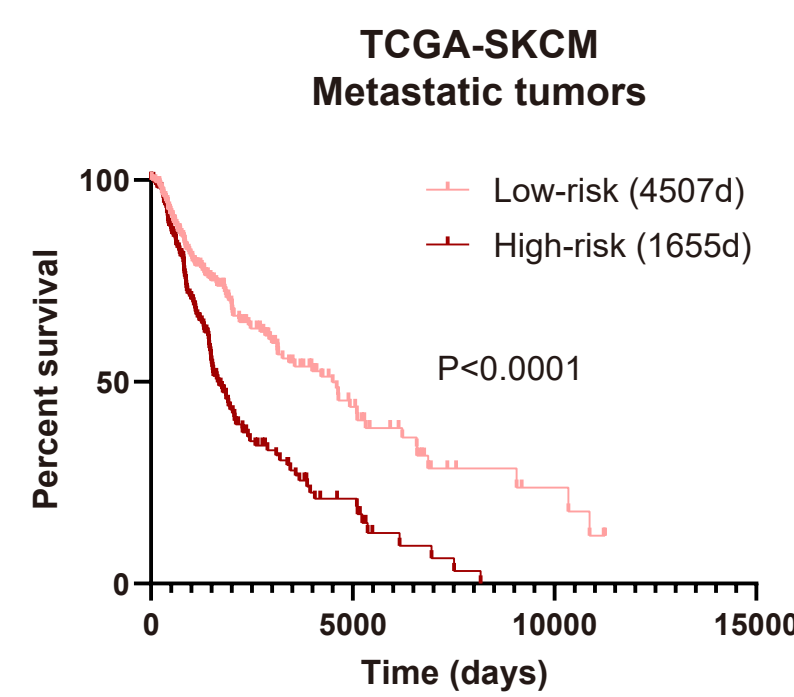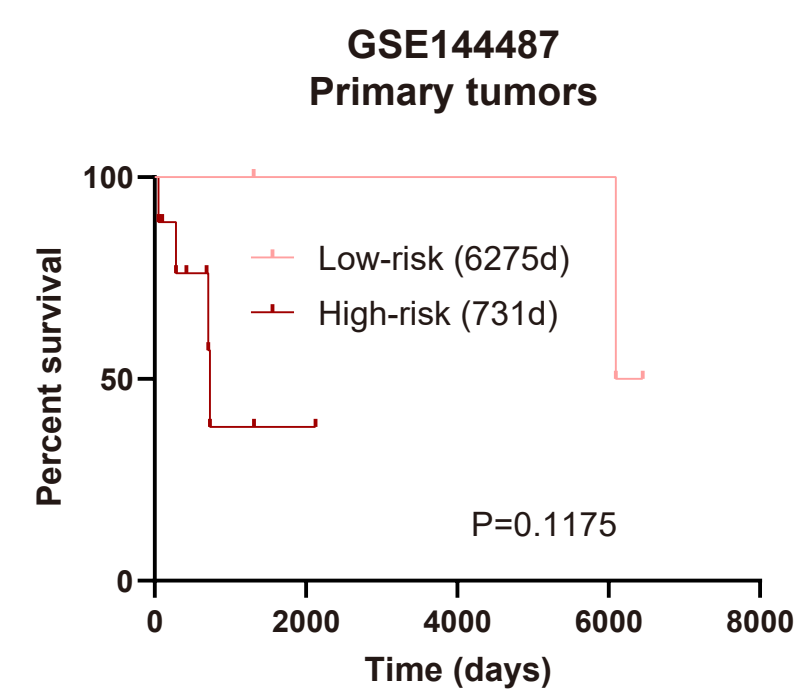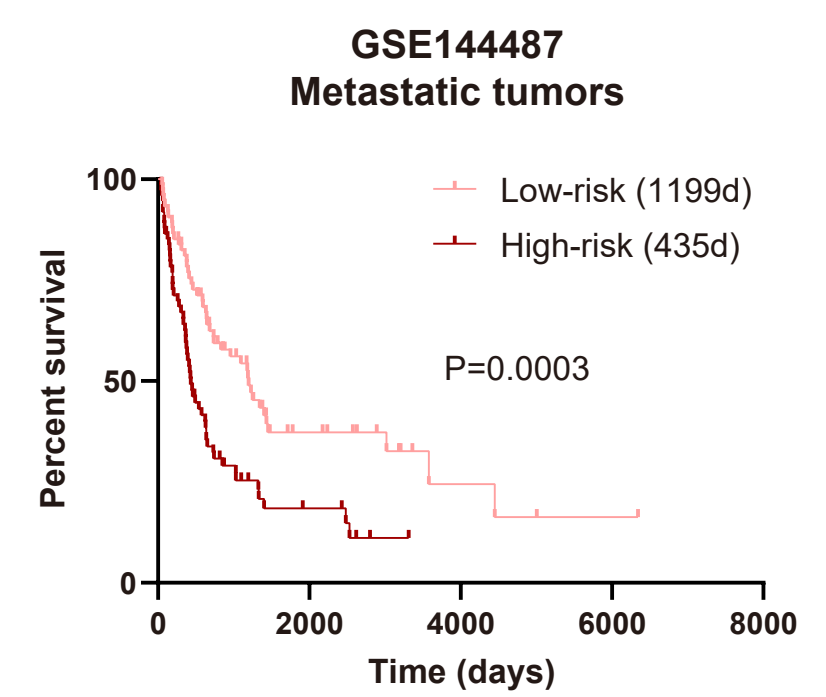

D

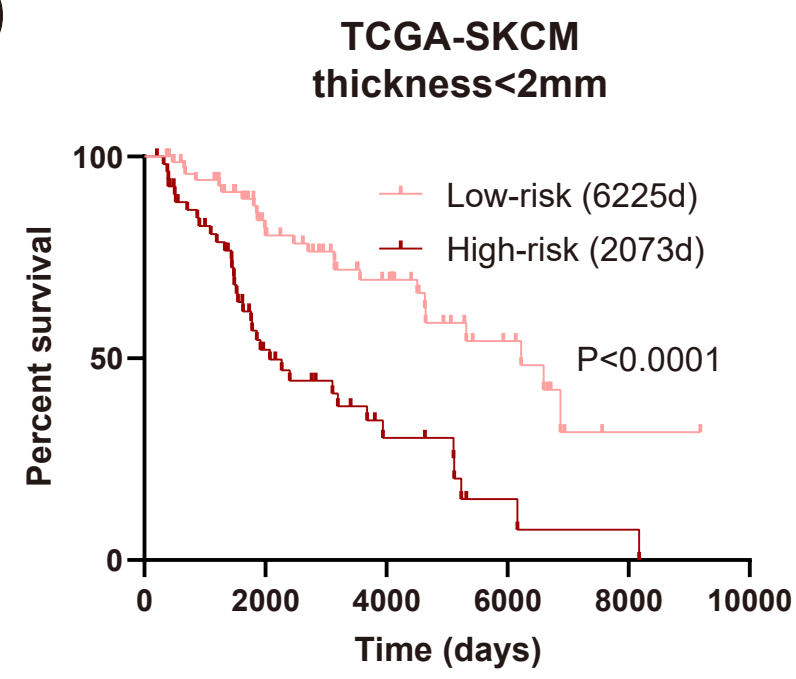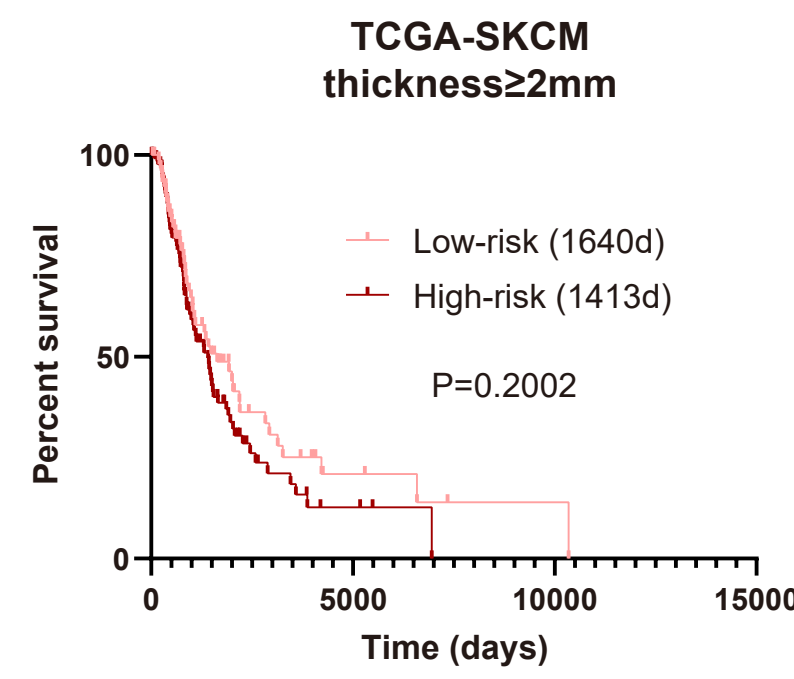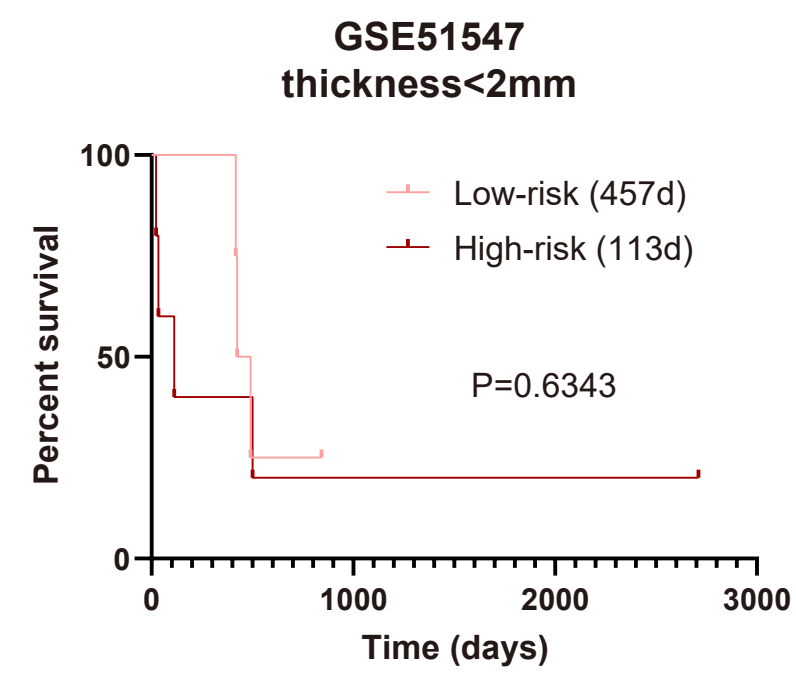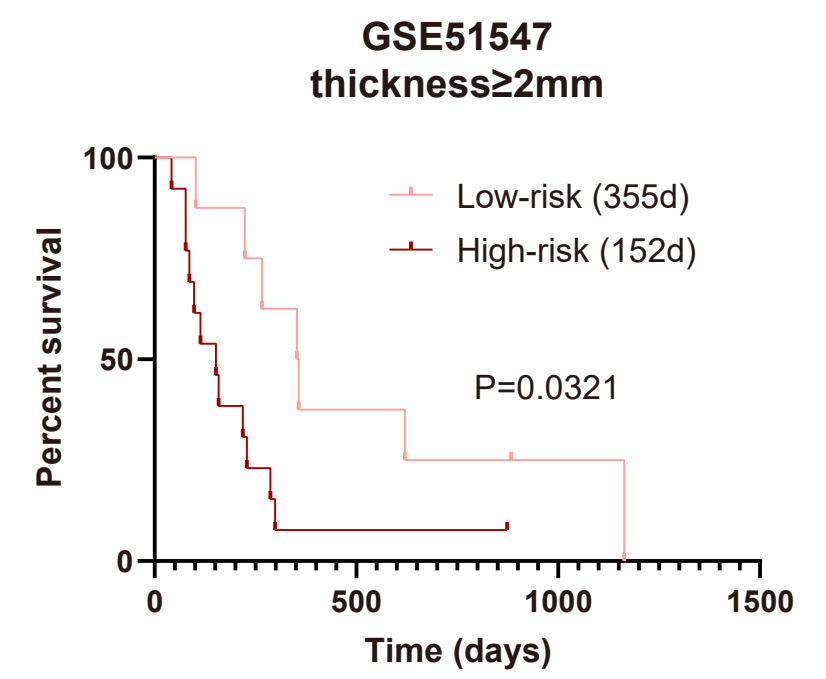

E

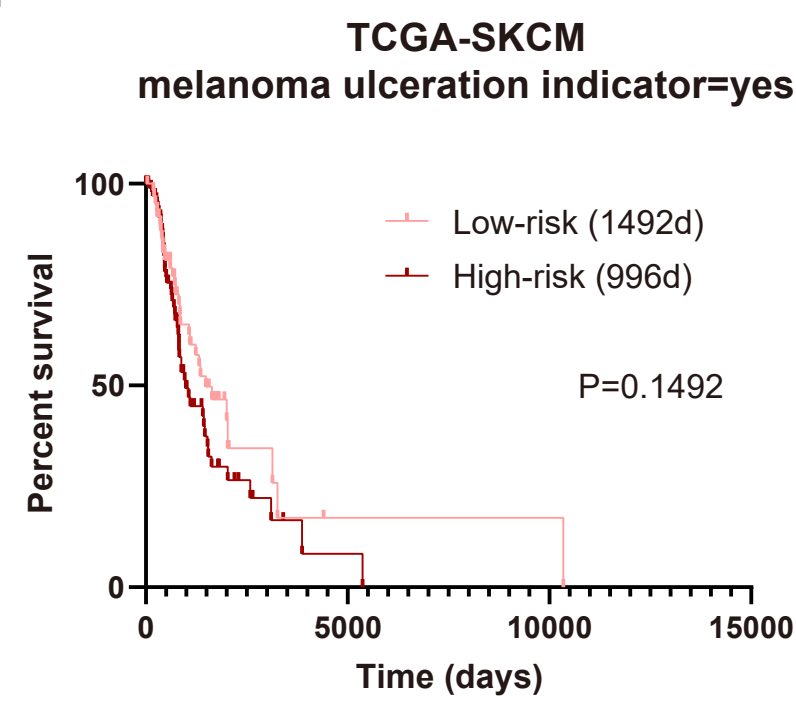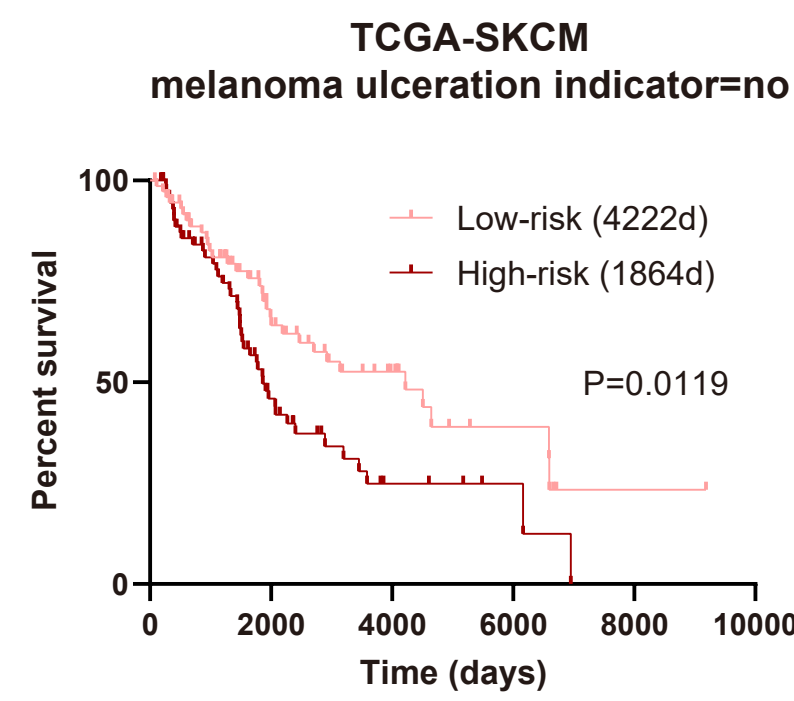

Supplement: Supplementary file 3 — Supporting information. [file IID3-12-e1331-s006.pdf]

A

TCGA-SKCM

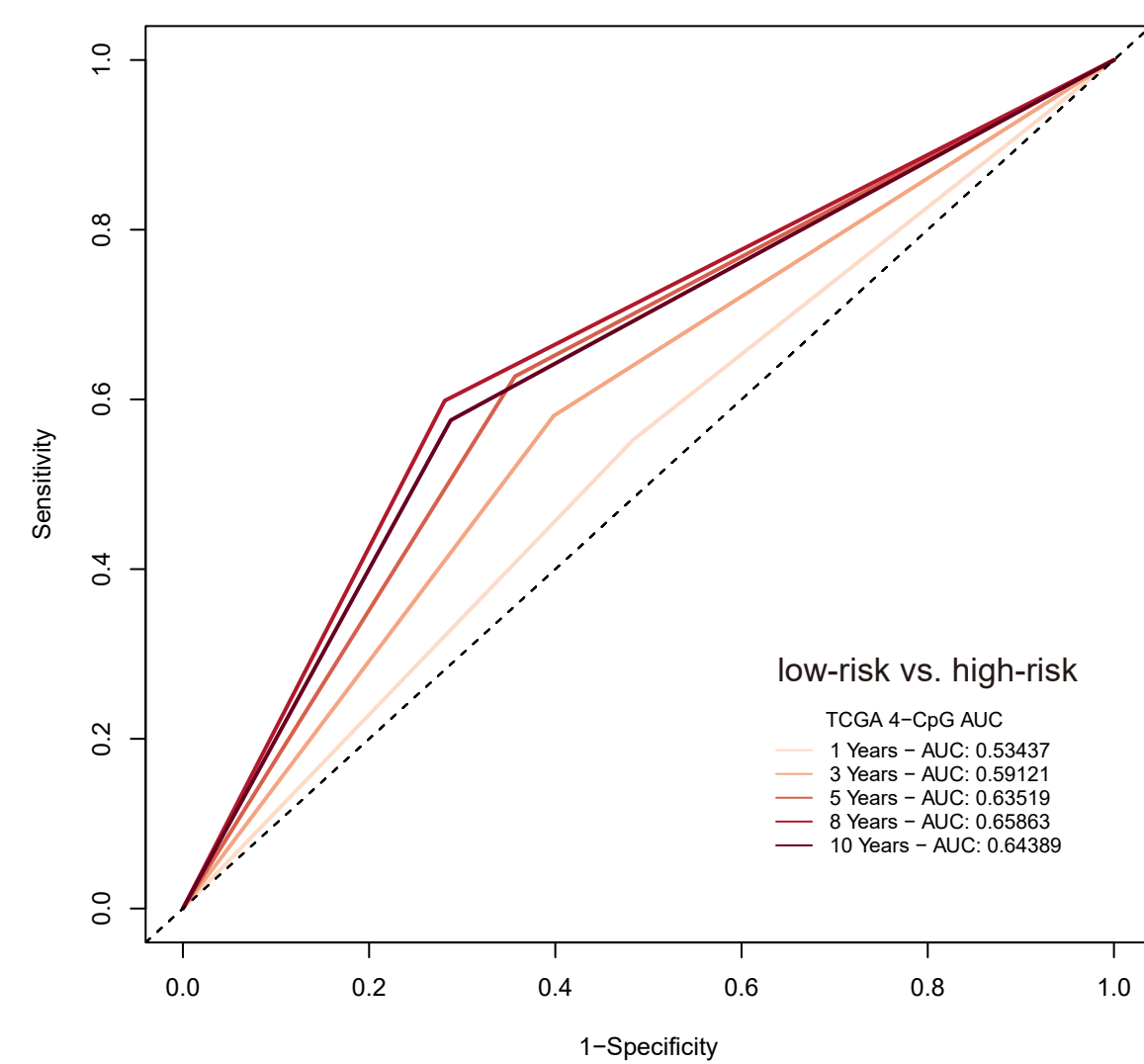

GSE144484

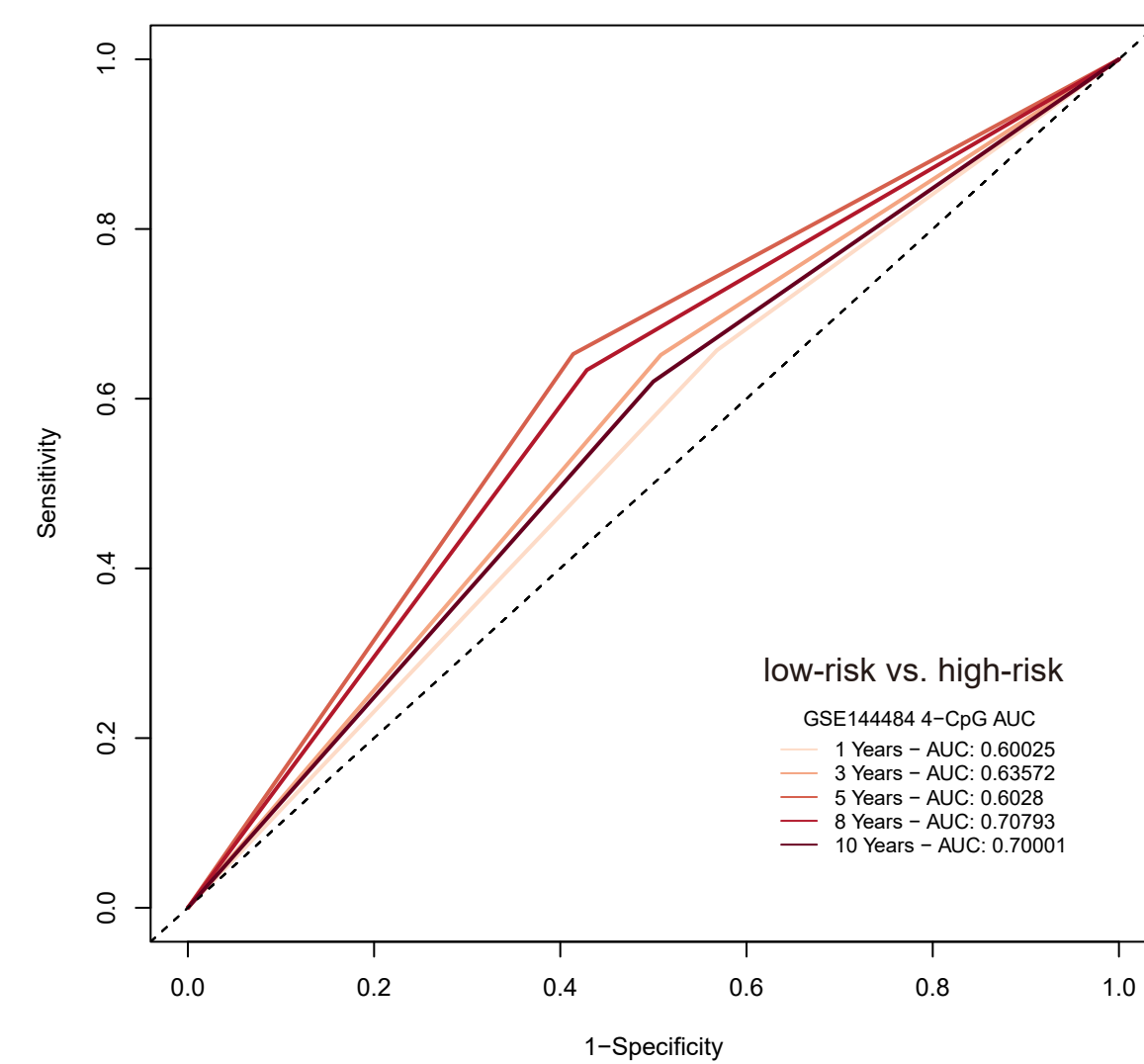

GSE51547

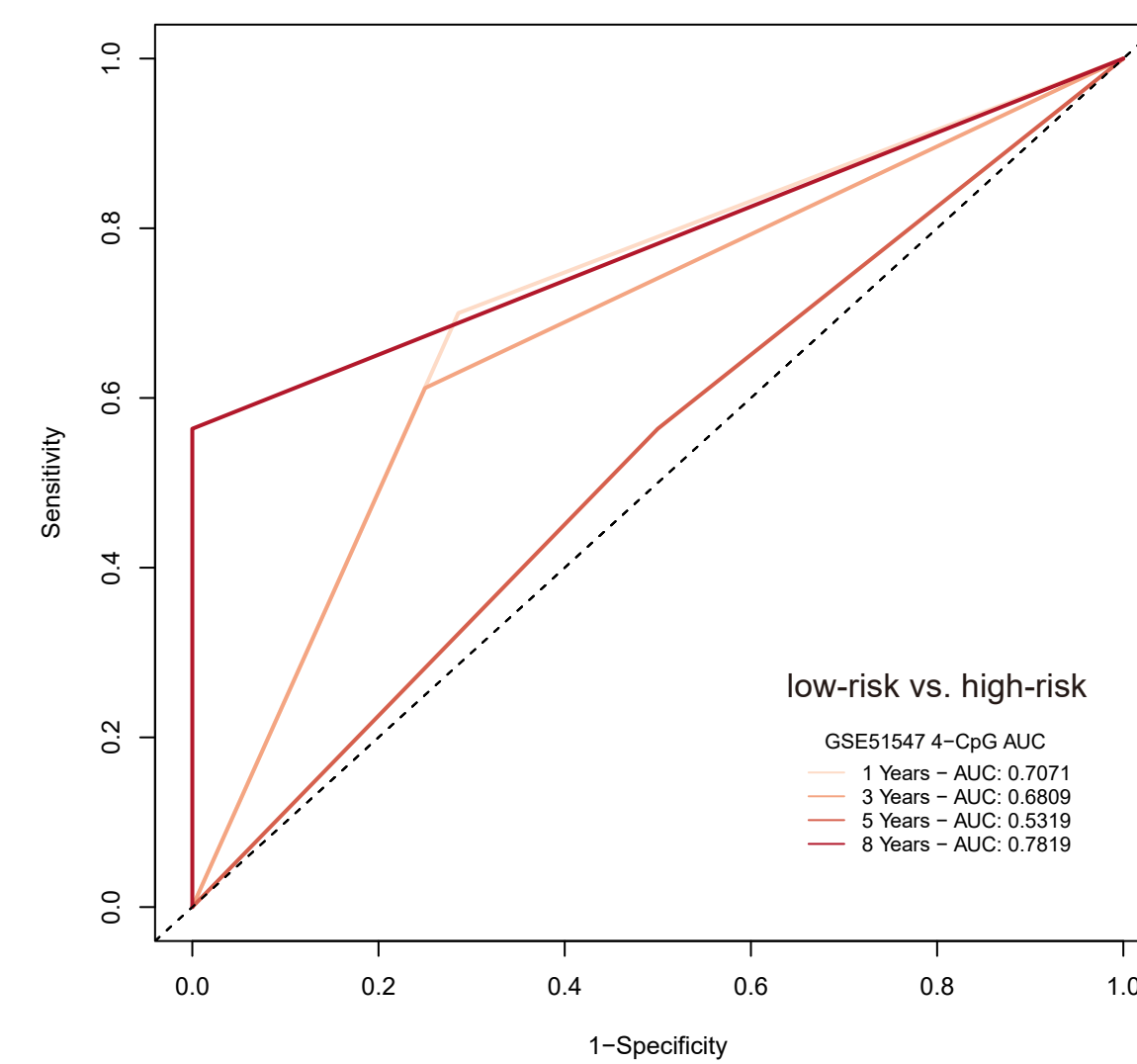

Meta-cohort

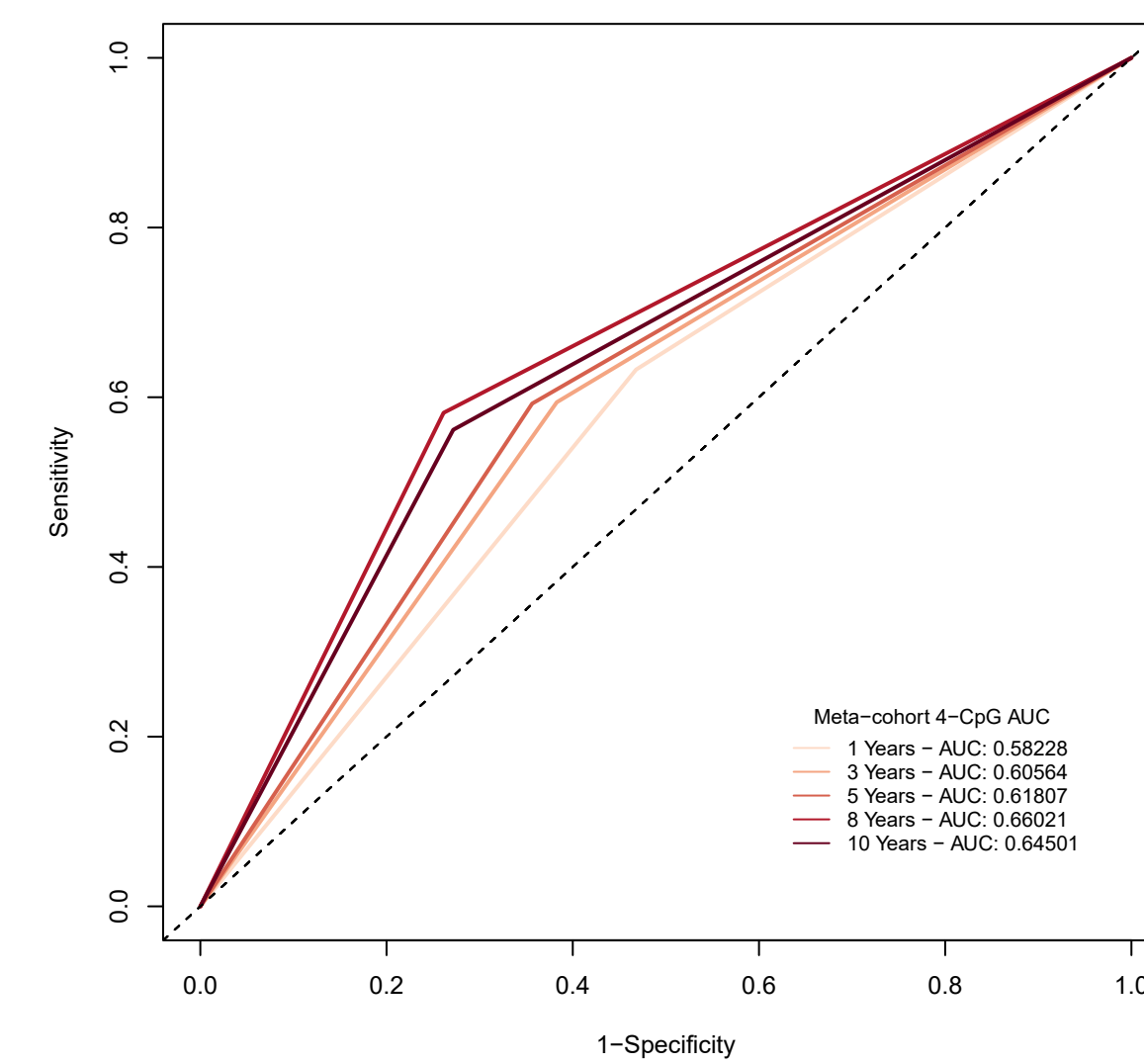

B

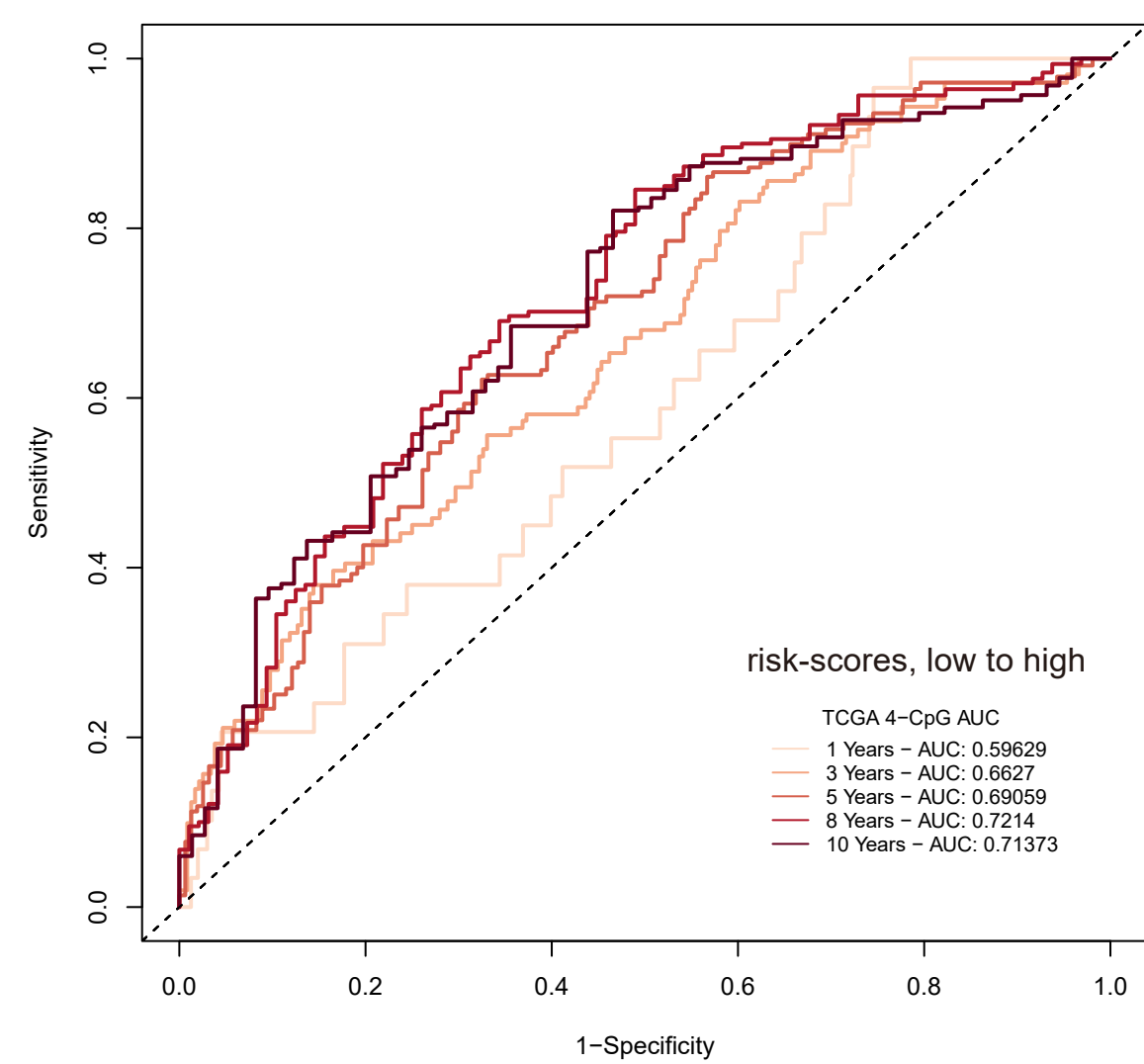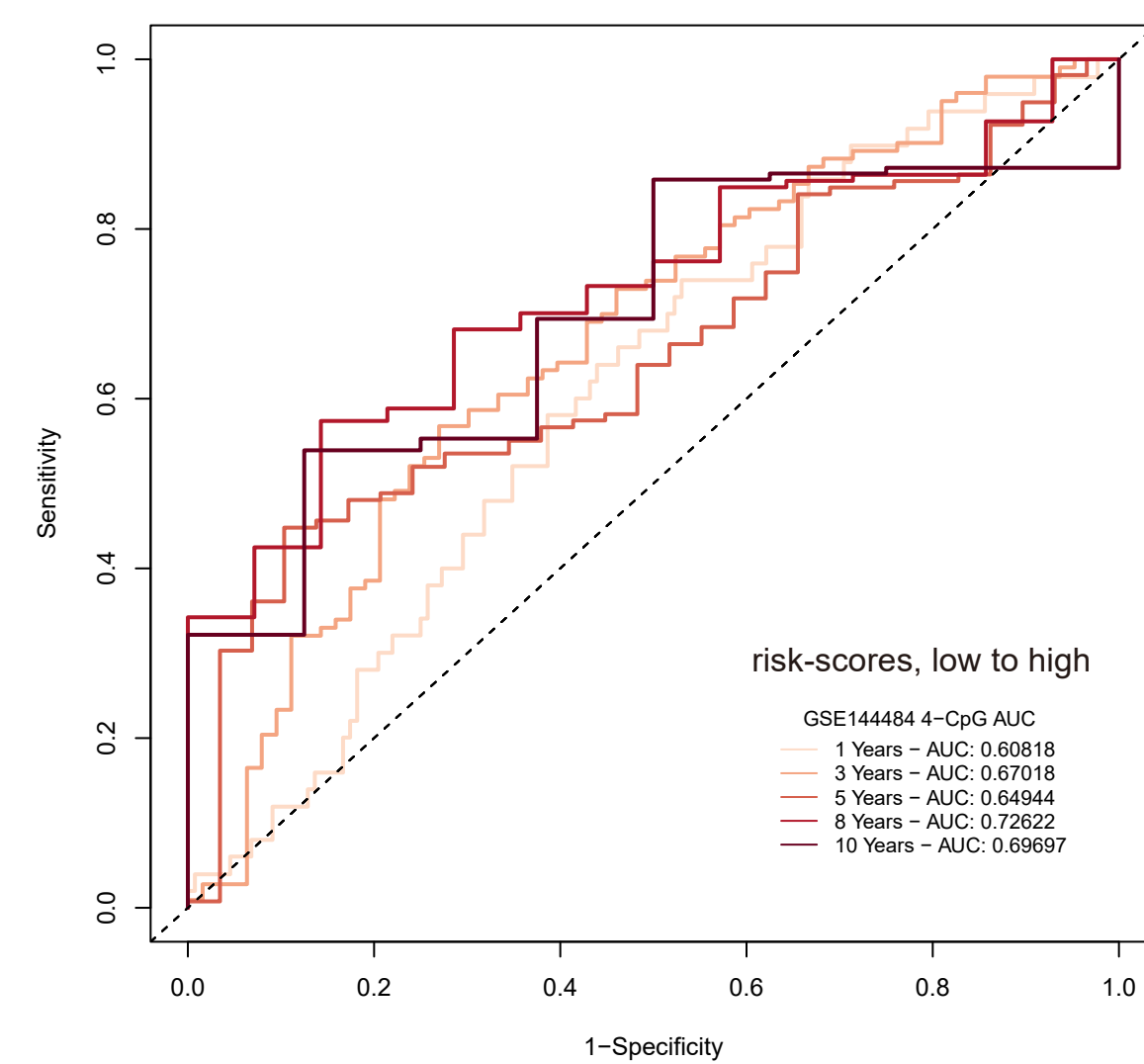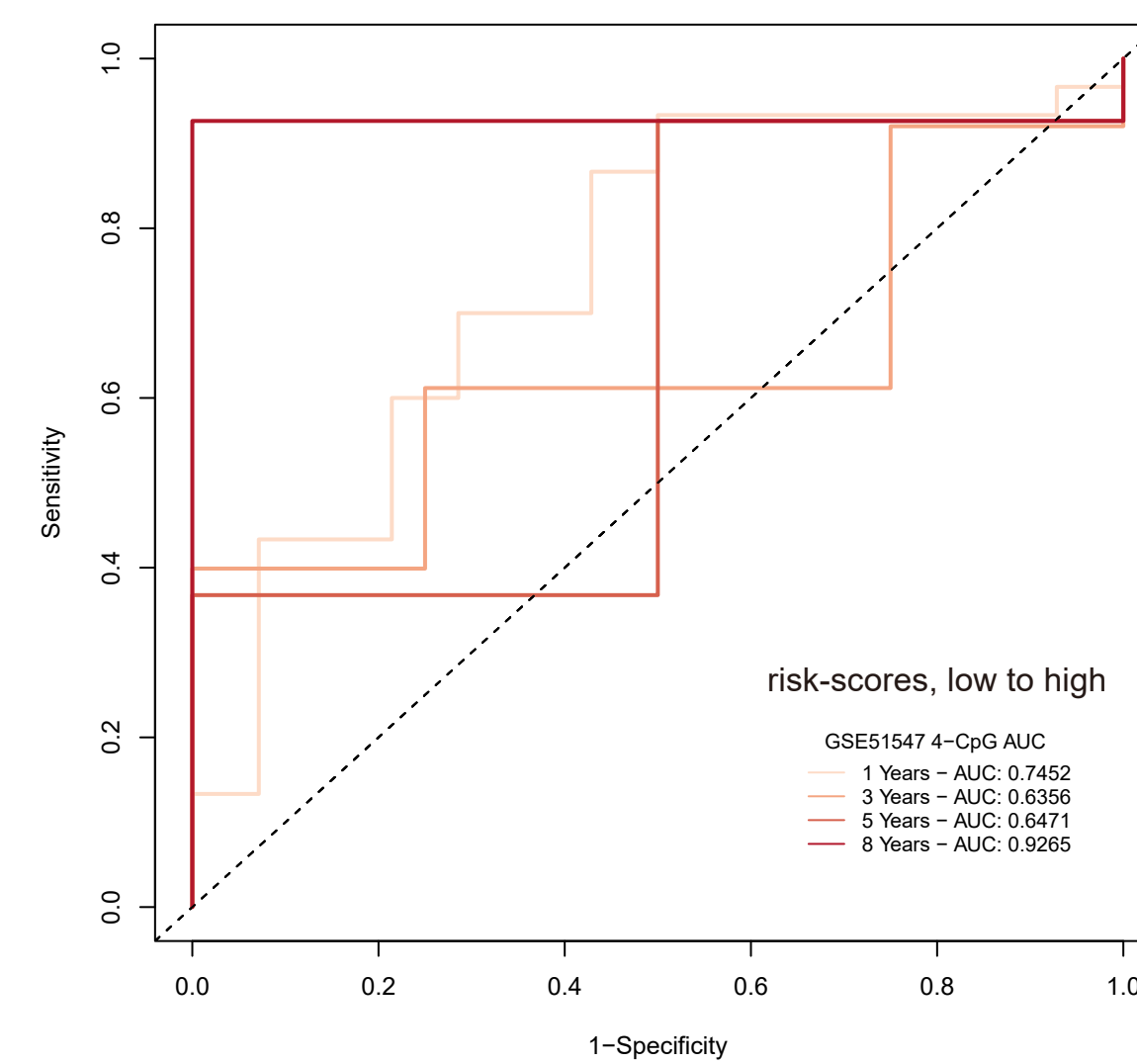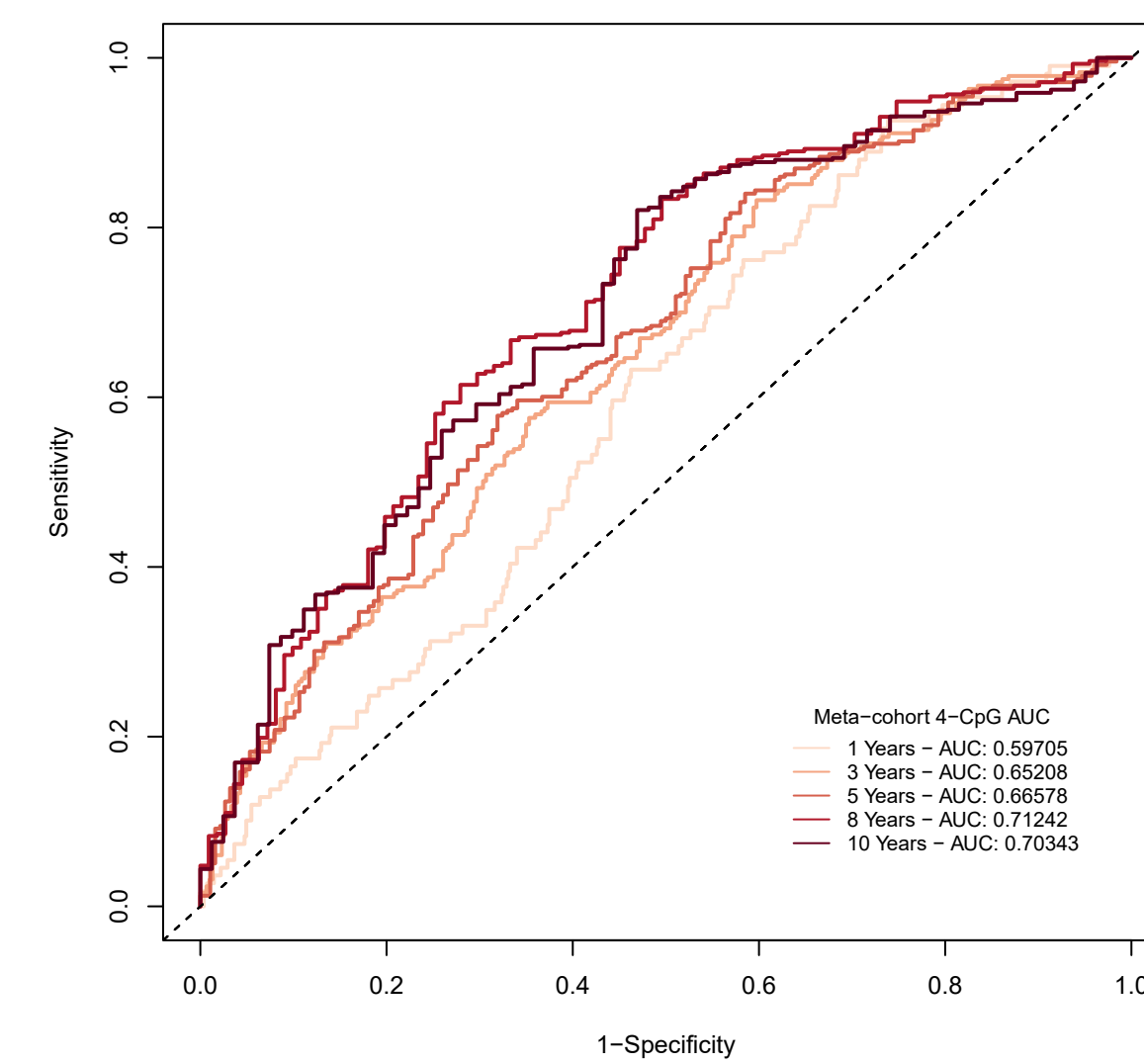

Supplement: Supplementary file 4 — Supporting information. [file IID3-12-e1331-s003.pdf]

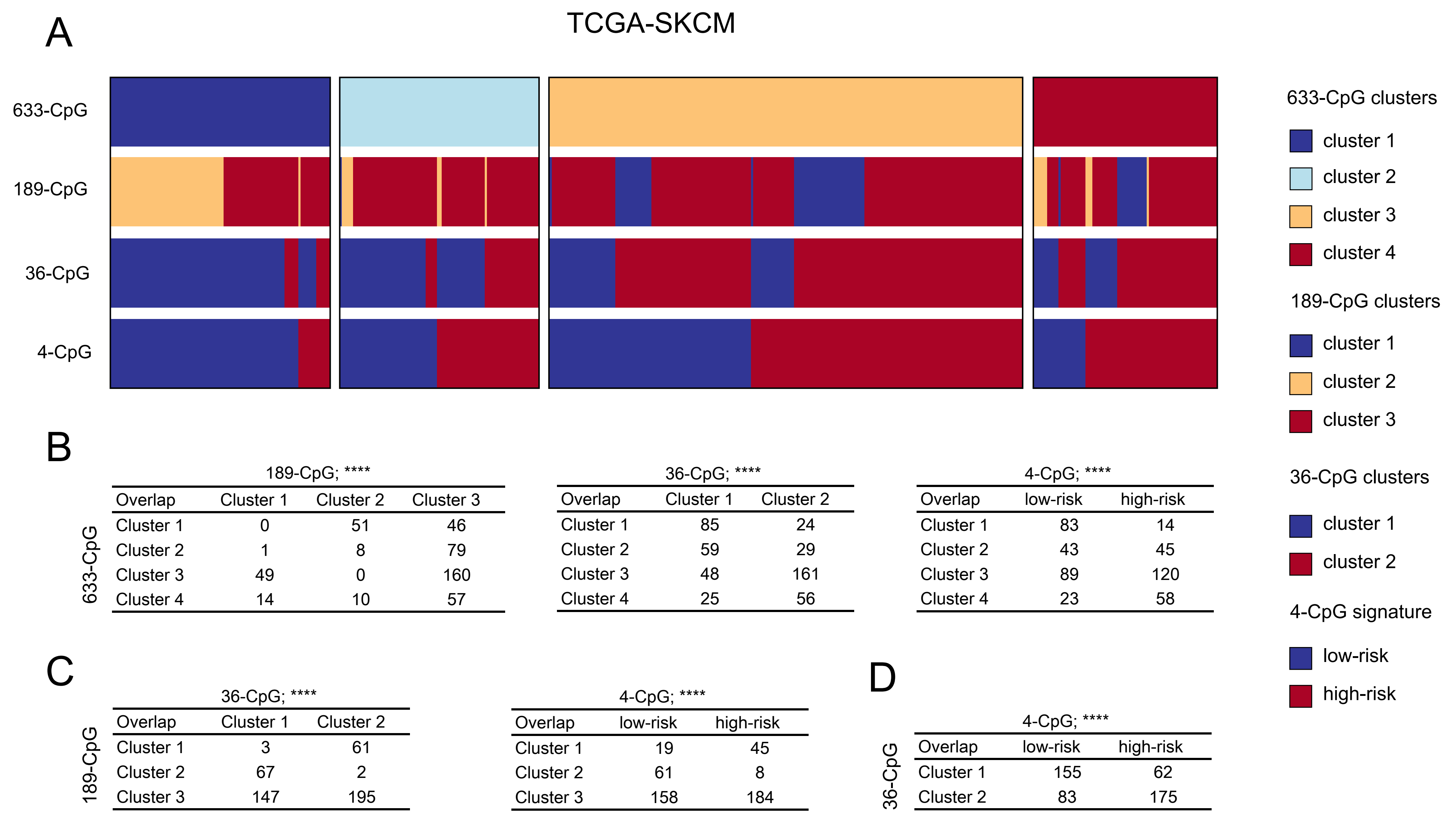

Supplement: Supplementary file 5 — Supporting information. [file IID3-12-e1331-s002.pdf]
