# Supplementary material for: DNA methylation variations of DNA damage response correlate survival and local immune status in melanomas
Source: Immun Inflamm Dis. 2024 Sep 10;12(9):e1331. doi: 10.1002/iid3.1331 (PMC11386344; doi:10.1002/iid3.1331)
Supplement: Supplementary file 6 — Supporting information. [file IID3-12-e1331-s005.docx]

**List of Supporting Information**

**Supplementary Figure S1: The prognostic performance of the 4-CpG risk score signature in stratified cohorts by clinical variables;** (A) Age < 60▒yrs vs. ≥ 60▒yrs; (B) Early stages (I or II) vs. Advanced stages (III or IV); (C) Primary vs. Metastatic tumors; (D) Thickness < 2▒mm vs. ≥ 2▒mm; (E) ulceration indicator yes vs. no.

**Supplementary Figure S2:** ROC-AUC of the 4-CpG signature in different patient cohorts; (A) low-risk vs. high-risk defined by median risk score value; (B) risk score values from low to high;

**Supplementary Figure S3: Correlations between the clusters defined by different CpG panels in TCGA-SKCM;** (A) heatmap for the clusters defined the 633-CpG, 189-CpG, 36-CpG and 4-CpG panel; (B) Comparison of the 633-CpG clusters with the clusters defined by the 189-CpG, 36-CpG and 4-CpG panels; (C) Comparison of the 189-CpG clusters with the clusters defined by the 36-CpG and 4-CpG panels; (D) Comparison of the 36-CpG clusters with the 4-CpG risk subgroups; statistical significance was indicated at the level of ns>0.05, *<0.05, **<0.01, ***<0.001 and ****<0.0001. ns=non-significant

**Supplementary Table S1:** **Characteristics of the selected CpGs related to DDR genes**

**Supplementary Table S2:** **Multi-variate Cox regression analysis of the 4-CpG risk score signature in each melanoma cohort**
